# Phosphorylation Site Dynamics of Early T-cell Receptor Signaling

**DOI:** 10.1371/journal.pone.0104240

**Published:** 2014-08-22

**Authors:** Lily A. Chylek, Vyacheslav Akimov, Jörn Dengjel, Kristoffer T. G. Rigbolt, Bin Hu, William S. Hlavacek, Blagoy Blagoev

**Affiliations:** 1 Theoretical Division, Los Alamos National Laboratory, Los Alamos, New Mexico, United States of America; 2 Center for Nonlinear Studies, Los Alamos National Laboratory, Los Alamos, New Mexico, United States of America; 3 Department of Chemistry and Chemical Biology, Cornell University, Ithaca, New York, United States of America; 4 Department of Biochemistry and Molecular Biology, University of Southern Denmark, Odense M, Denmark; 5 Department of Dermatology, Medical Center; Freiburg Institute for Advanced Studies (FRIAS); BIOSS Centre for Biological Signalling Studies; ZBSA Center for Biological Systems Analysis, University of Freiburg, Freiburg, Germany; 6 Department of Biology, University of New Mexico, Albuquerque, New Mexico, United States of America; University of Iowa, United States of America

## Abstract

In adaptive immune responses, T-cell receptor (TCR) signaling impacts multiple cellular processes and results in T-cell differentiation, proliferation, and cytokine production. Although individual protein–protein interactions and phosphorylation events have been studied extensively, we lack a systems-level understanding of how these components cooperate to control signaling dynamics, especially during the crucial first seconds of stimulation. Here, we used quantitative proteomics to characterize reshaping of the T-cell phosphoproteome in response to TCR/CD28 co-stimulation, and found that diverse dynamic patterns emerge within seconds. We detected phosphorylation dynamics as early as 5 s and observed widespread regulation of key TCR signaling proteins by 30 s. Development of a computational model pointed to the presence of novel regulatory mechanisms controlling phosphorylation of sites with central roles in TCR signaling. The model was used to generate predictions suggesting unexpected roles for the phosphatase PTPN6 (SHP-1) and shortcut recruitment of the actin regulator WAS. Predictions were validated experimentally. This integration of proteomics and modeling illustrates a novel, generalizable framework for solidifying quantitative understanding of a signaling network and for elucidating missing links.

## Introduction

Protein phosphorylation is a fundamental part of cellular information processing, with a role in controlling numerous physiological functions, including immune defenses [1]. Links between dysfunctional regulation of phosphorylation and disease underscore the need to elucidate underlying regulatory mechanisms [2]. To this end, phosphorylation-dependent signaling networks have been investigated extensively, largely in studies targeting individual proteins and interactions. However, cell signaling is marked by features, such as feedback and feedforward loops [3], [4], parallel pathways [5], and crosstalk [6], which may only be apparent when a network is studied as a whole. For this reason, multiplexed measurements of phosphorylation dynamics are needed, paired with reasoning aids for interpretation of these data.

A useful reasoning aid is a mechanistic model, meaning a model in which information about molecular interactions is cast in a form that enables simulations consistent with physicochemical principles. Simulation of such a model reveals the logical consequences of the collected knowledge upon which the model is based. Comparisons of model simulations to experimental measurements can drive discovery through generation of hypotheses and identification of knowledge gaps [7].

Successful integration of modeling and experimentation depends on both approaches having compatible and relevant levels of resolution. Phosphorylation dynamics can be elucidated using several high-throughput techniques, including reverse-phase protein arrays [8], micro-western arrays [9], and mass spectrometry (MS) [10]. MS-based techniques can yield quantitative information about the abundance of proteins phosphorylated at specific amino acid residues without reliance on availability of phosphosite-specific antibodies [11], and measurements can be made with fine time resolution [12], which is needed to decipher the order of phosphorylation events. Thus, MS-based proteomics has the potential to make unique contributions to systems biology modeling [13].

However, modeling and proteomics have not yet become tightly integrated, in part because of the technical challenges of constructing and parameterizing a model with sufficient detail and scope to be used for analysis of proteomic data. Proteomic measurements give information about phosphorylation levels at specific amino acid residues (sites); thus, a compatible model requires similar site-specific resolution. For this task, traditional modeling approaches (e.g., ordinary differential equations) can be difficult or impossible to apply [14], which has catalyzed development of the specialized techniques of rule-based modeling [15]. Rule-based models make it possible to simulate site-specific biomolecular interactions in a manner consistent with physicochemical principles.

Rule-based modeling has been used to study several immunoreceptor signaling systems [16], [17], [18], [19], [20], although in each case, the scope of the model has been restricted to a handful of signaling readouts. Development of models with sufficient scope to connect to proteomic data has faced additional challenges; large models can be costly to simulate and the complexity of the model can hinder communication of the model's content. To overcome these obstacles, simulation techniques for large models [21] and methods for model annotation and visualization [22] have recently been developed. Although these modeling capabilities have been demonstrated to a limited extent, use of large models to decode high-content data, generate hypotheses, and drive the discovery of biological insights has thus far remained uncharted territory.

We have developed a new approach for characterizing signal initiation using a rule-based model to interpret temporal phosphoproteomic data. We have applied this approach to study initiation of T-cell receptor (TCR) signaling, which is an essential process in the adaptive immune response [23]. The TCR and related antigen recognition receptors transmit signals that are dependent on site-specific details. These receptors are characterized by immunoreceptor tyrosine-based activation motifs (ITAMs), which each contain two tyrosine residues that can be phosphorylated. It has been found that the specific phosphoform of an ITAM can determine whether activating or inhibitory signals are transmitted [24]. Additionally, TCR signal initiation relies on the kinase LCK, which can be phosphorylated at a minimum of three sites: phosphorylation of two of these sites (Y394 and Y505) have opposing influences in regulating kinase activity [25], and phosphorylation of the third site (Y192) regulates the affinity of the SH2 domain [26]. These examples underscore the need to investigate the site-specific dynamics of immunoreceptor signaling [27].

Our results 1) characterize early TCR signaling with finer time resolution than previous proteomic studies of this system, 2) reveal mechanisms primarily operative on short timescales immediately after stimulation, and 3) demonstrate how mechanistic modeling and high-content measurements can be combined to develop a predictive understanding of cellular information processing.

## Results

### Immediate and extensive reshaping of the T-cell phosphoproteome

**Figure 1:**
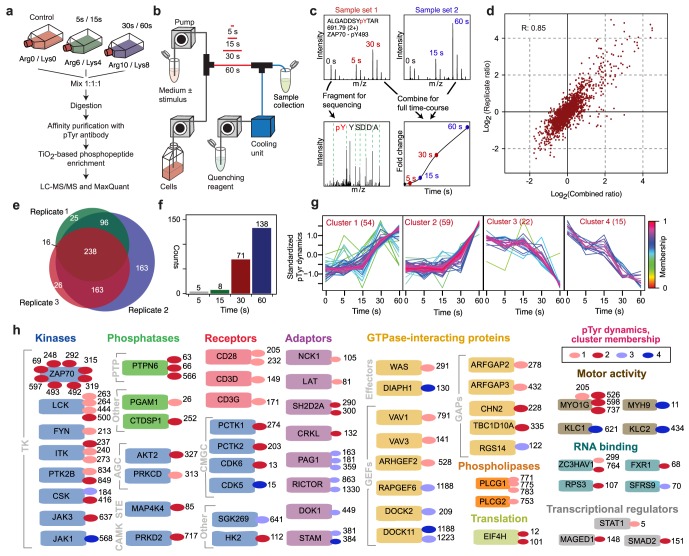
Phosphoproteomic analysis of TCR signal initiation. (A) T cells grown in SILAC media were stimulated with antibodies that crosslink CD3 and CD28. Lysates from differentially labeled cells were processed as indicated and relative abundances of phosphopeptides were quantified. (B) Flow system used to stimulate cells. (C) For each detected phosphopeptide, peak intensities in MS spectra were quantified to determine phosphorylation levels at 5, 15, 30 and 60 s after stimulation relative to the phosphorylation level in unstimulated cells. Results from paired spectra (top) were combined (bottom right). Peptides were identified by tandem MS (bottom left). (D) Measurement reproducibility. For each point, the *y*-axis indicates the relative phosphopeptide abundance measured in one of three replicate experiments; the *x*-axis indicates the corresponding average. *R* is Pearson's correlation coefficient. (E) Venn diagram indicating the numbers of phosphopeptides detected in individual and different combinations of replicate experiments. (F) Number of regulated pTyr sites (>2-fold change) at each indicated time point. (G) Results from clustering of time courses. (H) Diverse proteins undergo regulated phosphorylation. Boxes represent proteins; each oval and residue number next to a box identifies a regulated pTyr site and its cluster membership.

To characterize the first minute of TCR signaling, we performed time-resolved quantitative phosphoproteomic experiments, which allowed direct and accurate measurements of the changes in the levels of phosphorylation at individual tyrosine residues in response to TCR stimulation with anti-CD3, anti-CD28, and secondary antibodies (Fig. 1, A to C; see Materials and Methods). Three independent experiments were performed, resulting in the identification of over 700 unique pTyr sites, of which over 500 were detected in multiple experiments, with significant correlation across experiments (Fig. 1, D and E; Table S1 in File S1). Possible sources of variability in quantification and detection are mentioned in the Materials and Methods section. The level of phosphorylation for each site was quantified at 5, 15, 30 and 60 s of TCR/CD28 stimulation, relative to the corresponding level in unstimulated cells. These experiments targeted a period of signaling that has thus far been largely uncharacterized using MS-based proteomics or traditional biochemical assays, which have mostly been used at later timepoints. Our measurements map in unprecedented detail the earliest intracellular events and reveal that even within the first minute of TCR/CD28 co-stimulation, dramatic and diverse biochemical changes occur within the cell, preparing the ground for later events. To analyze these data, we took a knowledge-based/model-guided approach, which is summarized in Fig. S1A in File S2.

Regulated changes in phosphorylation (≥2-fold increases or decreases) occurred as early as 5 s after stimulation, with the number of regulated sites increasing to 138 after 60 s of stimulation (Fig. 1F). Time courses of phosphorylation fall into four distinct clusters, which reveal that the abundance of some phosphopeptides increase, others decrease, and some changes occur earlier than others (Fig. 1G). These results clearly demonstrate that even within the first 60 s of TCR stimulation there are diverse patterns of phosphorylation dynamics. Regulated sites map to proteins with various cellular functions, including pivotal signaling factors such as receptors, adapter proteins, phospholipases, phosphatases and kinases from multiple distinct kinase families. In the group of sites showing rapid dynamics we find well-established TCR signaling proteins such as LCK, LAT, PLCG1 among many others (Fig. 1H; Figs. S2 and S3 in File S2; Table S1 in File S1). These results attest to rapid, multi-functional signaling downstream of the TCR, consistent with the known diversity of pathways that emanate from the receptor [23].

Indeed, subsequent enrichment analysis (Fig. S2 in File S2) revealed that among the proteins with detected phosphorylation changes, the most frequent pathway association was with the TCR pathway. At the same time, other pathways, such as those influencing metabolism and protein synthesis, were also detected. These results suggest that TCR signaling may influence these general cellular functions quickly, consistent with evidence that T cells make committed decisions within 60 s of antigen contact [28].

### Dynamical modeling drives identification of knowledge gaps

**Figure 2:**
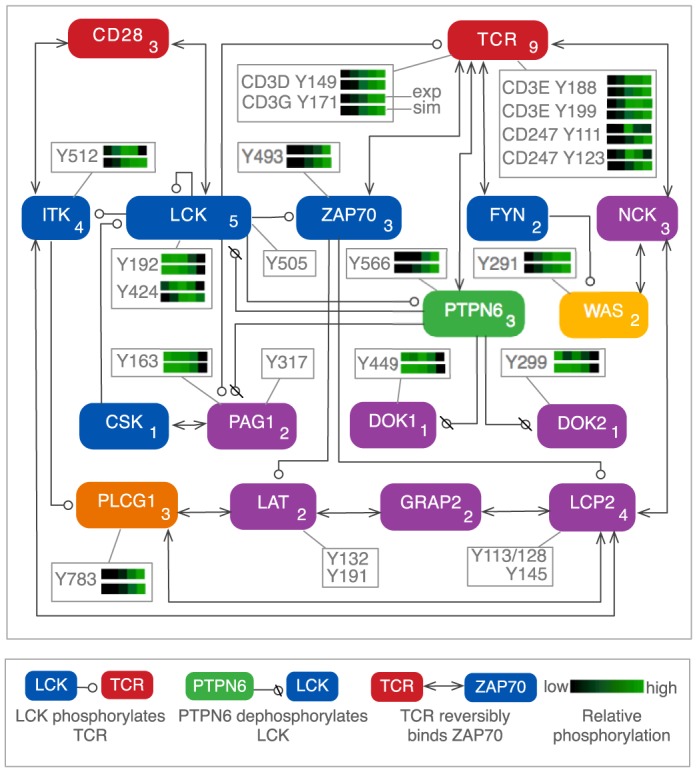
Model for TCR signaling. Proteins considered in a rule-based model for TCR signaling are represented by rounded boxes. Separate boxes indicate the phosphosites considered in the model. Sites detected in phosphoproteomic experiments are each associated with a pair of heatmaps, in which the upper heatmap reflects averaged experimental measurements of relative pTyr abundance and the lower heatmap reflects simulated phosphorylation levels at matching time points. The color scale of each heatmap is unique: black represents the lowest and green represents the highest level of phosphorylation for that site. Interactions are represented by arrows according to the conventions illustrated at bottom. The number in the lower right corner of a protein box represents the number of components of the protein (domains, motifs, and/or pTyr sites) considered in the model.

To investigate regulation of pTyr sites with well-characterized roles in TCR signaling, we developed a computational model based on principles of chemical kinetics and known protein-protein interactions (Fig. 2, Supplementary Text S1 in File S3, Fig. S4 in File S2, Table S2 in File S1, and Files A and B in File S1). The model, formulated in terms of local rules for interactions [15], accounts for 10 proteins containing 16 pTyr sites detected experimentally (Table S3 in File S2) and seven additional proteins linked to their regulation. The pTyr sites included in the model belong to three classes (Fig. S5 in File S2): 1) sites phosphorylated without dependence on prior receptor phosphorylation, 2) sites phosphorylated after receptor phosphorylation, and 3) sites that are dephosphorylated. Model-guided analysis of these pTyr site dynamics suggested that the phosphatase PTPN6 plays a positive role in TCR signaling (Fig. S1B in File S2) and that WAS is initially activated via a previously unappreciated pathway (Fig. S1C in File S2).

### Dephosphorylation of inhibitory pTyr sites

Involvement of a phosphatase in initiating TCR signaling was suggested by rapid dephosphorylation of six potentially inhibitory pTyr sites (Fig. 2 and Fig. S6 in File S2): 1) pY192 in the LCK SH2 domain, which reduces SH2-pTyr affinity [26]; 2) pY299 in DOK2, which binds RASA1 (p120 RasGAP), a negative regulator of RAS [29]; pY449 in DOK1, which binds CSK, which phosphorylates LCK and other SRC-family kinases (SFKs) at a C-terminal tyrosine residue to facilitate autoinhibition [30]; and 4) pY163, pY181, and pY417 in PAG1, which interact with SFKs to bring them into proximity of PAG1-bound CSK [31]. We also detected increased phosphorylation of Y566 in PTPN6, which is a substrate of LCK and is associated with positive regulation of phosphatase activity [32]. PTPN6 Y566 is phosphorylated as rapidly as ZAP70 Y493 (Fig. 2; cf. Fig. S6, G and K in File S2) and PTPN6 is the only protein tyrosine phosphatase that we observed to undergo regulated phosphorylation (Fig. 1H and Table S1 in File S1), suggesting a role in signal initiation. Incorporating PTPN6-mediated dephosphorylation of the sites listed above into the model enabled the model to reproduce measured time courses for these sites (Fig. 2 and Fig. S6 in File S2).

### Prediction and tests of a positive role for PTPN6 in early signaling

**Figure 3:**
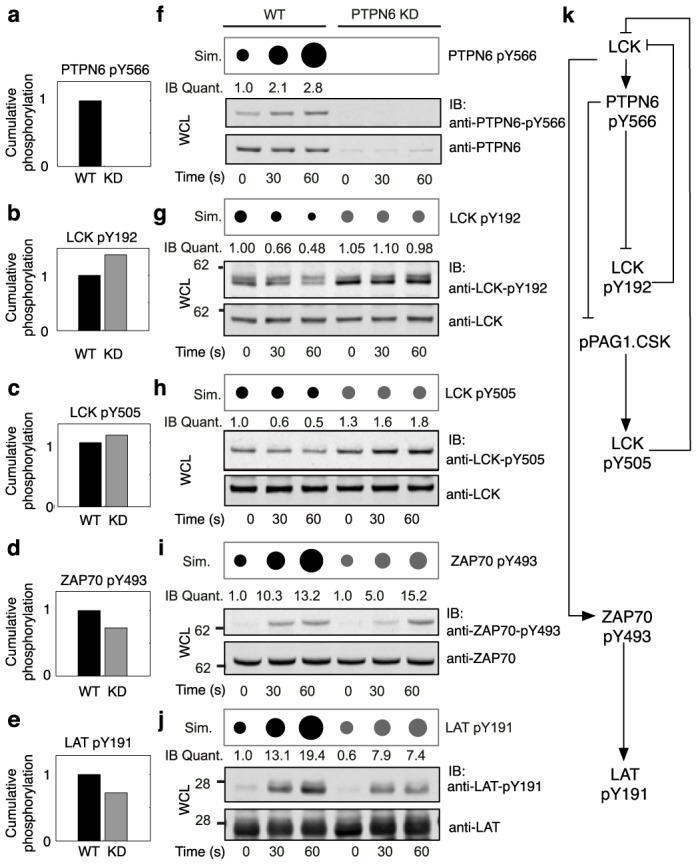
PTPN6 mediates positive feedbacks. (A to E) Model-predicted cumulative phosphorylation of the indicated pTyr sites in normal (WT) and PTPN6 KD cells. The cumulative phosphorylation of a site was calculated as the area under the corresponding time course of phosphorylation (0 to 60 s). Area is normalized to WT cells. (F to J) Simulation results (top) and immunoblots (bottom) showing the predicted and measured effects of PTPN6 KD on pTyr site dynamics. PTPN6 KD was modeled by setting the copy number of PTPN6 to 0. Simulated time courses are visualized as series of dots whose areas are proportional to relative phosphorylation levels. For each pTyr site, phosphorylation levels are normalized by the level of phosphorylation in unstimulated WT cells. Note that WT time courses present results shown previously in Fig. 2. IB, immunoblot; Quant., quantification; WCL, whole-cell lysate; Sim., simulation. (K) Hypothesized positive feedback loops involving PTPN6 incorporated in the model for TCR signaling. In these loops, LCK phosphorylates and activates PTPN6, and PTPN6 dephosphorylates sites that contribute to negative regulation of LCK. Thus, PTPN6 has a positive effect on phosphorylation events downstream of LCK, including LCK-mediated phosphorylation of ZAP70 and ZAP70-mediated phosphorylation of LAT. Blots are representative of the results from multiple (at least two) experiments. Each repeated immunoblot measurement is characterized by a coefficient of variation (CV) below 0.25, where CV is estimated as the ratio of the sample standard deviation to the sample mean.

We simulated the effect of lowered PTPN6 abundance. The model predicted dampening of stimulatory phosphorylation and enhancement of inhibitory phosphorylation, including increased phosphorylation of LCK Y192 (Fig. 3B), sustained phosphorylation of the C-terminal tyrosine (Y505) of LCK (Fig. 3C), decreased phosphorylation of Y493 in the activation loop of ZAP70 (Fig. 3D), and decreased LAT phosphorylation (Fig. 3E). According to the model, these simulation results arise from disruption of PTPN6-mediated positive feedback loops (Fig. 3K and Fig. S1, D and E in File S2).

To test the prediction that PTPN6 positively regulates TCR signaling, we used RNAi to knockdown PTPN6 (Fig. 3F, bottom). Expression of PTPN11 (SHP-2), a phosphatase that is closely related to PTPN6, was unaffected by PTPN6 knockdown, attesting to the specificity of the knockdown (Fig. S7 in File S2). We then used phosphosite-specific antibodies to monitor phosphorylation of LCK, ZAP70, and LAT in normal and PTPN6 KD cells after TCR/CD28 co-stimulation (Fig. 3, G and H). Sustained phosphorylation of LCK Y192 and Y505 (Fig. 3, G and H, bottom) and decreased phosphorylation of ZAP70 Y493 and LAT Y191 (Fig. 3, I and J, bottom) were observed, in qualitative agreement with model predictions (Fig. 3, B to E and G to J, top). At 60 s after stimulation, ZAP70 phosphorylation is similar in normal and PTPN6 KD cells (Fig. 3I, bottom), indicating that the positive early effect of PTPN6 on ZAP70 phosphorylation is transient. In contrast, the effect on LAT phosphorylation is evidently more sustained, as LAT phosphorylation in PTPN6 KD cells at 60 s is less than in normal cells.

To further test our model, we performed an *in vitro* phosphatase assay in which LCK was immunoprecipitated from PTPN6 KD cells and then incubated with purified recombinant PTPN6. We found that both Y192 and Y505 sites on LCK became dramatically less phosphorylated when incubated with PTPN6 compared to the mock treated sample (Fig. S8 in File S2). This finding is consistent with our model, in which pTyr sites observed to undergo net loss of phosphorylation, such as LCK Y192, are assumed to be substrates of PTPN6.

### A shortcut pathway connects the TCR to WAS activation

**Figure 4:**
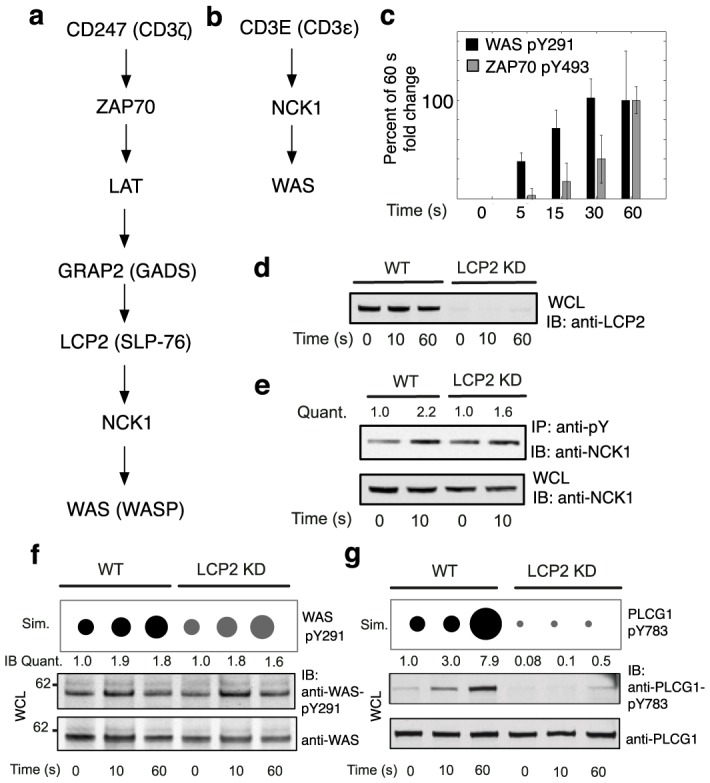
WAS activation. (A) Long pathway for WAS recruitment to the plasma membrane. Phosphorylated CD247 recruits ZAP70, which phosphorylates LAT. Phosphorylated LAT binds the GRAP2 SH2 domain. The GRAP2 SH3 domain binds LCP2. Phosphorylated LCP2, a substrate of ZAP70, binds the SH2 domain of NCK1/2. The C-terminal SH3 domain in NCK1/2 binds a proline-rich sequence (PRS) in WAS. (B) Short pathway for WAS recruitment. The N-terminal SH3 domain in NCK1/2 binds a PRS in CD3E, and the NCK1/2 C-terminal SH3 domain binds WAS. (C) Comparison of measured time courses of phosphorylation for WAS Y291 and ZAP70 Y493. Data is scaled such that the phosphorylation level of each site is 1 at 60 s. Error bars indicate standard deviations. (D) Efficiency of LCP2 KD. (E) Inducible association of NCK1 with pTyr-containing proteins in normal (WT) and LCP2 KD cells. (F and G) Simulations (top) and immunoblots (bottom) showing the predicted and measured effects of LCP2 KD on phosphorylation of WAS Y291 and PLCG1 Y783 upon TCR/CD28 co-stimulation for the indicated times. Simulation results are plotted as in Fig. 3, F to J. WT time courses present results shown previously in Fig. 2. Abbreviations are as in Fig. 3. Blots are representative of the results from multiple (at least two) experiments. As for Fig. 3, the estimated CV is less than 0.25 for each repeated immunoblot measurement.

A second novel mechanism of TCR signal initiation was suggested by fast phosphorylation of WAS Y291 (cf. Fig. S6, G and L in File S2). WAS can be recruited to the plasma membrane by the adaptor protein NCK1 through a pathway dependent on LAT and LCP2 (SLP-76) [33], which are substrates of ZAP70 [23] (Fig. 4A). However, we observed that WAS is phosphorylated before ZAP70 (Fig. 4C): the fold-change in WAS pY291 at 5 s is significantly greater than the fold change in ZAP70 pY493 (*p* = 0.019, one-tailed *t*-test). Thus, we reasoned that NCK1 may be present at the plasma membrane prior to ZAP70 activation, presumably through binding of its N-terminal SH3 domain to a proline-rich sequence (PRS) in CD3E, which takes place in the absence of TCR phosphorylation [34]. Adding this interaction to the model created a shortcut pathway to WAS activation (Fig. 4B) and enabled simulated phosphorylation of WAS to precede phosphorylation of ZAP70 (Fig. 2).

To confirm shortcut activation of WAS, we used RNAi to knockdown LCP2 (Fig. 4D), which mediates WAS activation as part of the well-characterized pathway of Fig. 4A. We found that the early association of NCK1 with pTyr sites is unaffected by LCP2 KD (Fig. 4E). Moreover, WAS phosphorylation is not substantially reduced in LCP2 KD cells (Fig. 4F), consistent with LCP2-independent phosphorylation of WAS. In contrast, phosphorylation of Y783 in PLCG1, which is dependent on LCP2 [23], is ablated in LCP2 KD cells (Fig. 4G).

WAS phosphorylation is dependent on LCP2 at times beyond the first minute of signaling [35], so we assume that the shortcut pathway to WAS activation is transient, which is consistent with the unusual signaling role of the CD3E PRS. Because this PRS overlaps Y188 in the CD3E immunoreceptor tyrosine-based activation motif (ITAM), phosphorylation of Y188 inhibits SH3-PRS binding and enables SH2-pTyr binding [36]. Model simulations indicate that the shortcut pathway to WAS activation is deactivated by ITAM phosphorylation (Fig. S9A in File S2) as the LCP2-dependent pathway is coordinately engaged (Fig. S9B in File S2). Engagement of LCP2-dependent pathways for WAS and PLCG1 activation is supported by immunoblot measurements of LCP2 phosphorylation (Fig. S9, C and D in File S2).

## Discussion

This study of pTyr site dynamics has revealed processes that have been systematically overlooked in the past because of the speed with which they occur. We have monitored the phosphosite dynamics of early TCR signaling with finer temporal resolution than in previous proteomic studies of TCR signaling (see Table S4 in File S2 and references cited therein) and with greater breadth than earlier studies of early TCR signaling events employing relatively low-throughput assays [37], [38], and we developed a mechanistic model for TCR signaling that reproduces measured time courses of phosphorylation for a greater number of specific sites than previously developed models for immunoreceptor signaling (see Table S5 in File S2 and references cited therein).

We detected over 100 pTyr sites that undergo greater than two-fold changes in abundance during the first minute of TCR signaling. Even on these short timescales, time courses show distinct patterns: the abundances of some pTyr sites increase, others decrease, and some changes occur sooner than others. The proteins containing these sites map to diverse cellular functions and include kinases, phospholipases, actin regulators, and transcription factors, many of which are known players in T-cell activation. The significance of these results is that by 60 s, which in many studies is taken as an early time point for measurement, significant changes have already occurred within the cell.

We found that multiple putatively negative regulatory sites (including sites in PAG1 and LCK) were rapidly dephosphorylated as the PTPN6 phosphatase was phosphorylated at an activating site. Inclusion of a mechanism causally linking these events allowed our model to reproduce measured time course data and to generate testable predictions. These predictions were validated experimentally, giving credence to the hypothesized link between PTPN6 activation and dephosphorylation of putatively inhibitory pTyr sites. Our model predicted that loss of PTPN6 would result in sustained phosphorylation of these pTyr sites, and reduction of phosphorylation at other, activating sites (including sites in ZAP70 and LAT). These predictions were confirmed through RNAi-mediated knockdown of *PTPN6* expression and immunoblot measurements with phosphosite-specific antibodies. These results provide strong motivation for future studies of the possible early positive role of PTPN6, ideally in primary cells. We note that a positive role for PTPN6 has been suggested by the results of earlier studies. For example, *in vitro*, PTPN6 has previously been found to be capable of dephosphorylating the inhibitory C-terminal tyrosine of LCK when the SH2 domain is deleted [39].

The view of PTPN6 as an overall negative regulator of TCR signaling [40] has been based mostly on studies of the *motheaten* mouse, which is deficient in Ptpn6 and suffers from severe autoimmunity [41]. Recent work has hinted at a more nuanced role. Studies of mice with a T-cell specific Ptpn6 deletion indicate that loss of Ptpn6 in T cells does not lead to overt autoimmunity [42], nor does it affect the number of memory precursor cells [43]. It has also been found that mechanisms controlling *PTPN6* expression are distinct from those controlling other negative regulators of TCR signaling [44]: levels of PTPN6 mRNA and protein are not affected by the miR-181a microRNA, which negatively regulates expression of multiple phosphatases linked to suppression of TCR signaling. Thus, PTPN6 appears to be somewhat enigmatic. Contributing to uncertainty about the function of PTPN6 is an incomplete catalog of its substrates, which is incomplete partly because known substrates do not match an obvious consensus sequence [45]. Our findings, together with those mentioned above, point to a need to identify signaling proteins whose phosphorylation states are regulated by PTPN6, and to characterize the function of this phosphatase in TCR signaling under precisely controlled conditions.

The results presented here suggest that, in Jurkat T cells, PTPN6 (the human ortholog of Ptpn6) has an early positive effect that accelerates signaling, before its negative effects become dominant. The negative effects of PTPN6, such as dephosphorylation of the LCK activation loop [25], may serve to prevent deleterious overshoots that would otherwise be caused by its positive effects, in addition to setting the baseline level of TCR signaling. As a participant in positive feedback loops, which can act as amplifiers, PTPN6 may also contribute to regulation of T-cell sensitivity. Such a role has been suggested in earlier studies of PTPN6 [32].

Several caveats are worth noting. Firstly, although we have demonstrated that PTPN6 acts directly on LCK Y192 and Y505 *in vitro*, we have not conclusively determined if PTPN6 directly acts on the sites that are observed to undergo dephosphorylation in our proteomics experiments, or if instead PTPN6 influences some or all of these sites in an indirect manner. Nonetheless, our knockdown results indicate that PTPN6 positively influences specific events in early signaling, and evidence for a more indirect mechanism would not alter this finding. Secondly, the dephosphorylated sites may have roles that are multifaceted, rather than strictly inhibitory. For example, phosphorylation of Y192 in LCK may enhance kinase activity by limiting SH2 association with the C-terminal phosphotyrosine that mediates autoinhibition, which is a regulatory mechanism that may be operative in the case of Src [46]. However, phosphorylation of LCK Y192 has been found to have an overall negative effect on important readouts of TCR signaling [26], indicating that impairing LCK's ability to associate with its binding partners outweighs potential enhancement of kinase activity through relief of autoinhibition. Thus, it is apparent that categorization of a protein or site as “positive” or “negative” is dependent on context and such categorization must be made with caution. Finally, the results presented here are based on the Jurkat T cell line, which has been a source of much of our current knowledge of TCR signaling mechanisms and is amenable to MS measurements. Use of a cell line was required to obtain the quantities of proteins required for MS-based assays of protein phosphorylation and to obtain the fine time resolution desired. However, Jurkat T cells do not perfectly recapitulate the behavior of T cells *in vivo*. Characterization of very early signaling mechanisms in primary T cells poses significant technical challenges and is beyond the intended scope of the present study.

The breadth and fine time resolution of our proteomic data allowed us to determine the order in which events occur. One of the fastest events observed was phosphorylation of the actin regulator WAS, which surprisingly preceded activating phosphorylation of the kinase ZAP70. It has previously been reported that WAS is recruited to the plasma membrane via a pathway involving LAT and LCP2 (SLP-76) [33], which are activated through ZAP70-dependent phosphorylation [23]. This mechanism of WAS activation did not allow our model to reproduce the observed WAS phosphorylation dynamics. In contrast, a previously unappreciated shortcut pathway, which is apparently active only transiently, allowed the model to reproduce the data. Experimentally, knockdown of *LCP2* expression did not attenuate the early WAS phosphorylation, consistent with model predictions and the presence of an alternative pathway. These results indicate that the flow of information through different pathways may shift as signaling progresses. Furthermore, the shortcut pathway may explain how the PRS in CD3E contributes to the ability of the TCR to respond to a range of agonist molecules. The PRS in CD3E and its interaction with NCK1 are known to be more consequential for responses to weak agonists [47] than strong agonists [48]. This difference may arise because weak agonists tend to induce only partial TCR phosphorylation [49], allowing longer-lasting NCK1-CD3E association. Although the interactions forming the shortcut pathway have been characterized individually, their combined role in facilitating rapid WAS activation has not hitherto been investigated. Thus, the results presented here complement past work by suggesting a potential mechanism by which the PRS of CD3E enables responses to weak agonists.

Our findings suggest that TCR signaling is initiated by proteins that transition from positive to negative roles. This strategy resembles bang-bang control [50], in which a controller assumes extreme values. PTPN6 appears to switch TCR signaling “on” upon signal detection and “off” after a period of signal transmission. Another apparent mediator of bang-bang control is CD3E, which is initially “on” and provides a shortcut pathway to WAS activation by recruiting NCK1 prior to receptor phosphorylation, but later is turned “off” as the CD3E ITAM is phosphorylated. The advantage of bang-bang control, or mode switching (a transition from positive to negative signaling by a protein with both on and off functions), can be appreciated by considering that a superior brake system decided the winner of the 1921 French Grand Prix by enabling fast approaches to turns [51]. The apparent on/off functions of PTPN6 and CD3E may allow a T cell to initiate signaling events with maximal acceleration and then avoid deleterious overshoots through application of a molecular brake.

Physiologically, we speculate that bang-bang control of TCR signal initiation may allow a T cell to launch rapid but controlled responses to infection. T cells scan antigen-presenting cells quickly and have been shown to decide if foreign antigen is present in under 1 min [28]. Thus, the effect of a fairly short delay in phosphorylation of LAT or WAS, for example, could potentially have a major impact on the number of antigen-specific T cells responding to an infection. Bang-bang control is operative in gene circuits with negative autoregulation [52] and in stem cell population dynamics [53] and may represent a widely used design principle of cellular regulatory systems.

## Materials and Methods

### Cell culture

Jurkat T cells, clone E6-1 (ATCC TIB-152), were grown in RPMI (Invitrogen) supplemented with penicillin/streptomycin (100 U/ml, 100 µg/ml), 10% dialyzed fetal calf serum (Invitrogen), and one of three SILAC labels (Sigma-Aldrich, Denmark): l-arginine and l-lysine (Arg0/Lys0); l-arginine-^13^C_6_-^14^N_4_ and l-lysine-^2^H_4_ (Arg6/Lys4); or l-arginine-^13^C_6_-^15^N_4_ and l-lysine-^13^C_6_-^15^N_2_ (Arg10/Lys8) (Fig. 1A). Before stimulation of TCR signaling, cells were serum starved for 16 h. Starved cells were diluted with medium (RPMI supplemented with 10 mM HEPES) to a density of 0.9–1.0×10^8^ cells/ml and stimulated with a 1∶1 mixture of pre-crosslinked anti-CD3 antibody (clone HIT3a, Santa Cruz) and anti-CD28 antibody (clone CD28.2, Santa Cruz) (4 µg/ml in RPMI supplemented with 10 mM HEPES). The anti-CD3 and anti-CD28 antibodies were crosslinked by incubation with anti-mouse IgG (Dako) at 4°C overnight. Differentially labeled cells (Fig. 1A) were stimulated for 0 s (Lys0/Arg0), 5 s (Lys4/Arg6), and 30 s (Lys6/Arg10) using a qPACE setup (Fig. 1B) as described earlier [12]. A second set of differentially labeled cells were stimulated for 0 s (Arg0/Lys0), 15 s (Arg6/Lys4), and 60 s (Arg10/Lys6) (2.7–3.0×10^8^ cells per condition). Because of the large number of cells required to obtain sufficient protein for LC-MS/MS analysis, we performed the three replicate temporal phosphoproteomic experiments of Fig. 1 serially over a time frame of months, which likely contributed to the measurement variability illustrated in Fig. 1D.

### Proteomics sample preparation

Cells were lysed in 8 M urea with 25 mM Tris, pH 8.0. Lysates from differentially labeled and stimulated cells (three conditions, as indicated in Fig. 1A) were mixed in a 1∶1∶1 ratio, centrifuged, and reduced with 1 mM DTT for 40 min at 25°C, followed by alkylation with 5.5 mM iodoacetamide for 40 min. Cell lysates were subjected to Lys-C (Wako) digestion at a 1∶100 enzyme/protein ratio for 5 h at room temperature. The lysates were diluted (4X) using 25 mM Tris, pH 8.0, and then digested with trypsin (Promega) overnight at room temperature. Digested cell lysates were acidified with TFA and desalted using Sep-Pak (Waters) in accordance with the manufacturer's instructions, followed by lyophilization of the tryptic peptides.

### Immunoprecipitation and purification of phosphopeptides

The lyophilized peptides were subjected to immunoprecipitation using a PhosphoScan Kit (P-Tyr-100, catalog number 7900, Cell Signaling Technology) and anti-phosphotyrosine antibody (clone 4G10, catalog number 16-101, Millipore). Briefly, peptides were dissolved in 2 ml of IAP buffer per experiment (50 mM MOPS, pH 7.2; 10 mM sodium phosphate; and 50 mM NaCl), refrigerated, centrifuged to remove undissolved peptides in the pellet, and immunoprecipitated with 80–100 µl of the anti-phosphotyrosine antibody resin for 3–4 h at 4°C. Beads were washed three times with the IAP buffer and two times with a salt solution (50 mM NaCl), and phosphopeptides were eluted using 0.15% TFA solution. Three sequential elutions were performed; each time, the volume of the 0.15% TFA solution used was equal to that of the bead volume. Eluted phosphopeptides were further purified by using TiO_2_ spheres as described earlier [10]. We note that it was not possible to use antibodies having the same lot numbers for each of the replicate temporal phosphoproteomic experiments of Fig. 1, which likely contributed to the measurement variability illustrated in Fig. 1D.

### LC-MS/MS analysis

Peptides were analyzed using LC-MS/MS as previously described [54]. Eluted samples were dried almost to completeness in a SpeedVac and analyzed using an LTQ-Orbitrap XL instrument (Thermo Scientific), which was interfaced with an Agilent 1100 nanoflow system (Agilent Technologies) and equipped with a nano-electrospray ion source (Proxeon Biosystems). Phosphopeptides were injected into a fused silica column packed in-house with 3 µm C18 beads (Reprosil, Dr. Maisch HPLC) applying a 120 min gradient from 8 to 64% acetonitrile in 0.5% acetic acid at a flow rate of 250 nl/min. We operated the Orbitrap XL in the data-dependent mode. The five most intense ions after full scan survey (MS spectra for *m/z* from 350 to 1,600) were subjected to MS/MS fragmentation using the CID activation technique with the following settings: *R* = 60,000 (MS resolution), a normalized collision energy of 35%, and an isolation window of 2.0 Th. In MS/MS acquisition, we used *q* = 0.25 (collision endothermicity) and an activation time of 30 ms. Slightly different settings were used for the third biological replicate: the range of *m/z* was 300–2000 and multistage activation (MSA) was used. For all MS runs, ions selected for fragmentation were dynamically excluded for 45 s and lock mass ions were used for internal mass calibration [55] to obtain constant mass accuracy during analysis. We note that sampling of ions for MS/MS analysis is stochastic in nature, which is likely to explain the variability in detection of peptides summarized in Fig. 1E. Detection of non-overlapping sets of peptides from experiment to experiment is expected unless coverage of the phosphoproteome is complete, which is difficult to achieve.

### Data analysis

Raw data files from three biological replicates were processed using MaxQuant (version 1.0.13.13) as described earlier [56]. Briefly, peak lists were generated by the MaxQuant program using the following search parameters: triple SILAC with heavy labels Arg6/Lys4 and Arg10/Lys8; a maximum of two missed trypsin cleavages; use of the six most intense peaks per 100 Da interval for generation of MS/MS peak lists; and a mass tolerance of 7 ppm on precursors and 0.5 Da (CID) for fragment ions. A fixed modification was carbamidomethylation of cysteine (Cys, +57.021464 Da) and variable modifications were oxidation of methionine (+15.994915 Da), N-terminal protein acetylation (N-terminal, +42.010565 Da), and phosphorylation of serine, threonine and tyrosine (Ser/Thr/Tyr, +79.966331 Da). We used the Mascot engine (v.2.3) (http://www.matrixscience.com) to search the generated peak-lists files (*.msm) against the IPI database (version 3.69) [57], which contains a list of frequently observed contaminants, concatenated with reverse copies of all entries. The acquired Mascot DAT files (*.dat) together with the raw files were processed and quantified by MaxQuant using the following parameters: the false discovery rate (FDR) for peptides, proteins and sites of modifications was required to be below 1% as assessed by the number of positive hits searched in the reverse database; and minimum peptide length was set at 6. MaxQuant automatically calculated the localization probabilities of all tyrosine phosphorylation sites as described earlier [10] and quantified intensity/peptide abundance ratios for each individual phosphosite. All tyrosine phosphorylated peptides (MS scan spectra) were manually inspected for arginine-proline conversion and each peptide abundance ratio was normalized in accordance with the number of proline residues in the corresponding peptide sequence. The peptide containing Y394 in LCK was matched to a miscleaved peptide that is unique to this protein.

### Bioinformatics analysis

For bioinformatics analysis, the peptide abundance ratios obtained from the three biological replicates were averaged. For cases where a pTyr site was detected in multiple peptides, we considered the least modified peptide when evaluating pTyr site dynamics. Clustering of pTyr sites showing ≥2-fold dynamics at a minimum of one time-point was performed using the fuzzy *c*-means algorithm as implemented in GProX with default parameters [58]. Enrichment analysis was also performed using GProX by retrieving Gene Ontology (GO) [59] and Pfam [60] annotations from the UniProt database [61] and testing for over-representation of terms in each cluster, which was assessed using Fisher's exact test. Only terms occurring at least two times in a cluster and attaining a *p*-value less than 0.05 after correction for multiple testing using the Benjamini and Hochberg algorithm were regarded as significant. For identification of enriched pathways, UniProt accession keys were uploaded to DAVID [62] and analyzed with default parameters. Sequences of kinase domains were aligned using Clustal W2 [63] and the resulting tree file was visualized using the iTOL tool [64]. The “princomp” function of MATLAB was used for principal component analysis.

### Modeling and simulation

A chemical-kinetics model for TCR signaling in a single cell was formulated using a rule-based approach, which enabled concise representation of biomolecular interactions and efficient simulation of multi-site phosphorylation [15]. The goal of model building was to leverage available mechanistic knowledge to construct a model that includes as many observed pTyr sites as possible. The knowledge base of the model was developed through a data-guided literature search. Phosphorylation sites and the proteins containing these sites were selected for inclusion in the model if they were detected in the phosphoproteomic experiments, were known to be involved in TCR signaling based on information in the primary literature, and if they had a known kinase, phosphatase, and/or binding partner. A second set of proteins and sites were included if, based on published findings, they were necessary for regulation of the sites detected in experiments. Residue numbers were assigned for naming purposes in accordance with standard UniProt numbering. The proteins and interactions included in the model are identified and discussed in the Supplementary Text S1 in File S3. The initial model that we constructed on the basis of available mechanistic knowledge was deemed deficient because it was unable to reproduce the observed dephosphorylation dynamics of four putatively inhibitory pTyr sites (Fig. S6I, M–O in File S2) and also because it was unable to reproduce the observed fast phosphorylation dynamics of WAS (Fig. S6L in File S2). To address these shortcomings, we extended the model to include the hypothetical mechanisms of Fig. 3K (Fig. S1B, D, E in File S2) and Fig. 4B (Fig. S1C in File S2). Unlike other aspects of the model, these mechanisms cannot be supported by literature citations, which is why experimental tests of model predictions focused on probing these aspects of the model. The mechanism of Fig. 3K was initially suggested by detection of activating phosphorylation of PTPN6 (Fig. 1H; Fig. S6K in File S2). The mechanism of Fig. 4B was initially suggested by reports in the literature about the interactions that comprise the shortcut pathway to WAS activation, especially the interaction between CD3E and NCK1 [34]. Rules for noncovalent interactions and post-translational modifications (i.e., tyrosine phosphorylation and dephosphorylation) were specified using BNGL, a domain-specific language for formulating models of biochemical reaction kinetics [65]. BioNetGen [65] was used to process File A in File S1 (a.bngl file) to generate an XML encoding of the model, which served as input for NFsim [21], together with File B in File S1 (a.rnf file). File B in File S1 specifies simulation protocols, including an equilibration procedure that served to find the unstimulated steady state. NFsim implements a particle-based kinetic Monte Carlo algorithm [66]. Thus, NFsim produces results that reflect the stochastic nature of chemical reactions; it uses rules to generate reaction events, which are selected to occur randomly. For this reason and also because our model is formulated for a single cell, whereas our experimental measurements correspond to averages over a large population of cells, we performed multiple simulation runs and the results were averaged to obtain smooth curves for comparisons with the proteomic, population-averaged data. NFsim simulation results were validated using a different simulation tool, RuleMonkey [67]. Model parameters were estimated in three ways, as indicated in Table S2 in File S1 (see the footnotes). Some parameters were assigned values reported in the literature, which were determined in one of two ways: in an experimental study or in an earlier modeling study. Other parameters were determined through simplifying assumptions or physicochemical constraints. These parameters were assigned values related to and determined by other parameter values; the relationships between the independent and dependent parameters are given in Table S2 in File S1. Finally, some parameters were determined through fitting. These parameters were constrained during fitting in one of two ways. Some were simply constrained to have positive values. Others were constrained to have values between specified lower and upper bounds, which were set on the basis of various empirical considerations, which are noted in Table S2 in File S1. Fitting was performed initially using a brute force approach (coarse grid search), followed by targeted parameter refinement using the variable metric method [68]. *PTPN6* and *LCP2* knockdowns were modeled by setting the copy numbers of PTPN6 and LCP2 to zero. The model visualization in Fig. S4 in File S2 is drawn in accordance with established conventions [22]. Simulation results were visualized using MATLAB, version 7.10.0 (R2010a) (MathWorks, Natick, MA).

### DNA constructs for RNAi silencing and generation of stable cell lines

A lentiviral vector pSicoR (Addgene plasmid 11579) [69] and plasmids of the 3rd generation packaging system for producing viral particles [70] were used: pMD2.G (Addgene plasmid 12259), pMDLg/pRRE (Addgene plasmid 12251) and pRSV-Rev (Addgene plasmid 12253), which were obtained via Addgene's Material Transfer Agreement. These DNA plasmids were kindly deposited in Addgene by Drs. Tyler Jacks and Didier Trono.

An EGFP cassette in the vector pSicoR was exchanged with a puromycin resistance gene cassette, resulting in a modified pSicoR-puro vector allowing puromycin resistance-based selection of shRNA expressing cells. RNAi sequences potentially targeting the *PTPN6* and *LCP2* transcripts were generated using available Web resources (http://www.dharmacon.com) according to published recommendations for siRNA/shRNA design [71], [72]. shRNA DNA constructs were designed using recommended guidelines [69] and available Web resources. Briefly, the shRNA sequences were synthesized (DNA Technology, Denmark) as two complementary DNA oligonucleotides:

5′-T(N19)TTCAAGAGA(rN19)TTTTTTC-3′ and

5′-TCGAGAAAAAA(N19)TCTCTTGAA(rN19)A-3′

where N19 is the sense strand of the target sequence and rN19 is the antisense strand. The oligonucleotides were annealed as described earlier [72] and directly cloned into the vector pSicoR-puro. Clones were selected for verification by DNA sequencing. We used the following targeting sequences for RNAi: 5′-GAGCATGACACAACCGAAT-3′ for *PTPN6* and 5′-GGACCAGACAGAAGAGAGA-3′ for *LCP2*. Verified DNA constructs were used to produce lentiviral particles as described earlier [69] with modifications. Briefly, 10 µg of lentiviral vector and 5 µg of each packaging plasmid were co-transfected in one 15 cm dish of HEK-293T cells using the transfection reagent METAFECTENE (Biontex Laboratories) according to the manufacturer's instructions. Supernatants were harvested 48 and 72 h after infection and viral particles were concentrated by ultracentrifugation at 115,000 RCF for 2 h at 4°C. Viral stocks were diluted in cell culture media and used for infection of Jurkat T cells to generate stable cell lines expressing the described RNAi constructs. Cells stably expressing shRNA sequences were grown in RPMI medium with 4 µg/ml of puromycin for 5 days and used for qPACE-based co-stimulation of TCR/CD28 signaling for immunoblot experiments. Depletion of PTPN6 and LCP2 was confirmed by immunoblotting.

### Immunoblotting

Equal amounts of normal Jurkat T cells (WT) and Jurkat T cells with stable knockdown of *PTPN6* (PTPN6 KD) or *LCP2* (LCP2 KD) were stimulated for either 0, 30, and 60 s or 0, 10, and 60 s using our qPACE setup (Fig. 1B). Harvested cells were lysed using ice-cold lysis buffer [modified RIPA buffer: 150 mM NaCl; 50 mM Tris, pH 7.5; 1% v/v NP-40; 1 mM EDTA; proteases inhibitors (cOmplete Tablets, Roche); 1 mM sodium orthovanadate; 2 mM NaF; and 2 mM β-glycerophosphate]. The cell lysates were centrifuged, mixed with 6× Laemmli buffers and resolved on Novex 4–12% Bis-Tris gradient gels (Invitrogen) using MES running buffer followed by protein transfer to nitrocellulose membrane, blocking with 5% BSA and incubation with primary and HRP-conjugated secondary antibodies. To quantify western blots, we used the Analyze Gels function in the ImageJ software tool (http://imagej.nih.gov/ij/docs/guide/user-guide.pdf). Chemiluminescence was measured, and we considered different exposure times to ensure that images were analyzed well before saturation. Values for bands corresponding to site-specific antibody staining were normalized using values for corresponding total protein loading controls. The following antibodies were used for western blots: Phospho-Lck (Tyr 505), Phospho-Zap-70 (Tyr 493), Phospho-LAT (Tyr 191), Zap-70, WASP, NCK1 (Cell Signaling); LAT (Santa Cruz Biotechnology, Inc.); Phospho-WASp (Tyr 290) (Sigma-Aldrich); Phospho-Lck (Tyr 192) (Abcam); and Shp-1, Shp-2, and Slp-76 mouse mAb (BD Biosciences). Secondary anti-mouse and anti-rabbit antibodies were obtained from GE Healthcare, UK.

### In vitro dephosphorylation assay

Jurkat T cells with stable knockdown of *PTPN6* were starved for 16 hours and harvested by centrifugation. The cells were lysed using ice-cold lysis buffer [modified RIPA buffer: 150 mM NaCl; 50 mM Tris, pH 7.5; 1% v/v NP-40; 1 mM EDTA; proteases inhibitors (Complete tablets, Roche); 1 mM sodium orthovanadate; 2 mM NaF; and 2 mM β-glycerophosphate]. The cell lysate was centrifuged; a supernatant was supplemented with the SDS up to 0.5% and incubated for 30 minutes on ice. The cell lysate was diluted with the lysis buffer up to 0.1% SDS. Mouse anti-LCK antibody (6 µg) bound to Protein G beads was used for immunoprecipitation of LCK for 5 hours at 4°C. The beads were washed three times with the lysis buffer and five times with ice-cold *in vitro* assay buffer (50 mM HEPES, pH 7.4, 2 mM DTT, 100 mM NaCl, 2 mM EDTA). Washed beads were divided into two equal parts with 50 µl *in vitro* assay buffer. The first sample was supplemented with 1 µg of an active human recombinant protein PTPN6 (Millipore, cat. No. 14-591) and second sample was vehicle treated. Both samples were incubated at 37°C for 30 minutes with gentle shaking. Thereafter, the samples were mixed with 6× Laemmli buffers and resolved on Novex 4–12% Bis-Tris gradient gels (Invitrogen) using MOPS running buffer followed by protein transfer to nitrocellulose membrane, blocking with 5% BSA and incubation with primary and HRP-conjugated secondary antibodies.

For immunoprecipitation, an anti-LCK antibody (Mouse, clone MOL 171, BD Pharmingen) was used. For immunoblotting, antibodies specific for phosphorylated Y192 in LCK (Abcam) and LCK (Rabbit, Cell Signaling) were used.

### Supporting Information

The Supporting Information consists of 17 items: Tables S1 and S2 and Files A and B in File S1; and Figures S1–S9 and Tables S3–S5 in File S2; and File S3 (Supplementary Text S1). Table S4 in File S2 includes citations of References [73], [74], [75], [76], [77], [78], [79]. Table S5 in File S2 includes citations of References [80], [81], [82], [83], [84], [85]. The Supplementary Text S1 (File S3) includes citations of References [86], [87], [88], [89], [90], [91], [92], [93], [94], [95], [96], [97], [98], [99], [100], [101], [102], [103], [104], [105], [106], [107], [108], [109], [110], [111], [112], [113], [114], [115], [116], [117], [118], [119], [120], [121], [122], [123], [124], [125], [126], [127], [128], [129], [130], [131], [132], [133], [134], [135], [136], [137], [138], [139], [140], [141], [142], [143], [144], [145], [146], [147], [148], [149], [150], [151], [152], [153], [154], [155], [156], [157], [158], [159], [160], [161], [162], [163], [164], [165], [166], [167], [168], [169], [170], [171], [172], [173], [174], [175], [176], [177], [178], [179], [180], [181], [182], [183], [184], [185], [186], [187], [188], [189], [190], [191], [192], [193], [194], [195], [196], [197], [198], [199], [200], [201], [202], [203], [204], [205], [206], [207], [208], [209], [210], [211], [212], [213], [214], [215], [216], [217], [218], [219], [220], [221], [222].

## Supporting Information

File S1 **This Zip file combines Tables S1 and S2 (Excel spreadsheets) and Files A and B (plain-text files).** **Table S1 in File S1**. Proteomic data. This Excel spreadsheet provides a listing time courses and residue numbers of phosphopeptides detected in each of three LC-MS/MS experiments. **Table S2 in File S1**. Parameter estimates. This Excel spreadsheet provides a listing parameter estimates used in the model for TCR signaling. **File A in File S1**. Executable model specification. This plain-text file provides an executable model specification, which can be processed by BioNetGen. The filename extension should be changed to “.bngl” for processing by BioNetGen. **File B in File S1**. Simulation protocol. This plain-text file provides a definition of a simulation protocol, which can be processed by NFsim. The filename extension should be changed to “.rnf” for processing by NFsim.
(ZIP)

File S2 **This PDF file combines Figures S1–S9 and Tables S3–S5.** **Figure S1 in File S2**. Overview of methodology and summary of main results. (**A**) An integrated experimental and model-based approach was used to characterize initial phosphorylation events in TCR signaling, generate non-trivial predictions, and test these predictions. A model based solely on previously elucidated mechanisms of TCR signaling did not reproduce the phosphorylation dynamics observed for the following five sites: LCK Y192, DOK1 Y449, DOK2 Y299, PAG1 Y417, and WAS Y291. Incorporation of novel mechanisms enabled the dynamics of these sites to be reproduced and led to generation of predictions that were tested experimentally. (**B**) Proposed roles of PTPN6 in early and late signaling. In early signaling (bold lines), PTPN6 plays a positive role by dephosphorylating negative regulatory sites, including LCK Y192, PAG1 Y163, DOK1 Y449, and DOK2 Y299. Later in signaling (thin lines), the negative regulatory capabilities of PTPN6 become dominant. (**C**) Proposed dual pathways for activation of WAS. In early signaling (bold lines), WAS is recruited to the plasma membrane through interaction with NCK1/2 in association with CD3E. As signaling progresses over time, the longer pathway for WAS recruitment (thin lines), which is dependent on LCP2, becomes dominant. (**D**) A PTPN6-mediated positive feedback loop in which PTPN6 dephosphorylates LCK Y192, thereby enhancing the ability of the LCK SH2 domain to interact with pTyr sites. LCK activates PTPN6 through phosphorylation and direct interaction. (**E**) In a second PTPN6-mediated positive feedback loop, LCK phosphorylates and activates PTPN6. PTPN6 dephosphorylates PAG1, which reduces the ability of PAG1 to co-localize LCK and CSK, which reduces phosphorylation of LCK at its inhibitory C-terminal tyrosine and relieves autoinhibition. **Figure S2 in File S2**. Enrichment analysis. For proteins containing regulated pTyr sites, we tested for enrichment of associated GO terms compared to proteins containing detected but unregulated pTyr sites (i.e., pTyr sites for which phosphorylation changed less than two-fold). (**A**) Cluster-specific enrichment analysis based on “biological process” terms. (**B**) Cluster-specific enrichment analysis based on “molecular function” terms. (**C**) Cluster-specific enrichment analysis based on “cellular compartment” terms. (**D**) We also tested for enrichment of associated Pfam domain names. In A through D, color is used to indicate the negative logarithm (base 10) of the *z*-transformed *p*-value associated with each term. The lighest shade of green corresponds to the highest level of enrichment. Black corresponds to no enrichment. (**E**) Information about detected pTyr sites was uploaded to the DAVID resource and processed using default parameters to identify pathways enriched for regulated pTyr sites. The *y*-axis reports the negative logarithm (base 10) of the *p*-value for each of the indicated pathways. Pathway enrichment scores are reported on the *y*-axis. The most enriched pathway is the “T cell receptor signaling pathway.” **Figure S3 in File S2**. Phylogenetic relationships of protein kinases with detected pTyr sites. The tree shown was built based on kinase domain sequences. Protein kinases that contain regulated pTyr sites are indicated with red lettering; these kinases are also represented in Fig. 1h. Kinase families are indicated by background colors. The following abbreviations are used for protein kinase family names: TK, tyrosine kinases; TKL, tyrosine kinase-like; CMGC, the CDK/MAPK/GSK3/CLK group; AGC, protein kinase A, G, and C families; CAMK, calcium and calmodulin regulated kinases; and STE, homologs of the yeast STE7, STE11, and STE20 genes. **Figure S4 in File S2**. Visualization of model. Proteins, domains, and linear motifs are represented as boxes, which are nested to indicate structural relationships. Lines that begin and end with an arrowhead represent direct binding interactions. Arrowheads point to functional components that mediate protein-protein interactions. Lines that originate at a box representing an enzyme (a kinase or phosphatase) and end with an open circle, or open circle overlayed with a diagonal bar, indicate enzyme-substrate relationships. An open circle denotes phosphorylation and an open circle overlaid with a diagonal bar denotes dephosphorylation. Flags (vertical lines connected to a small square box at top and a text label at bottom) represent sites of post-translational modification. All of these sites are pTyr sites. Compartmental locations of proteins are indicated by boxed labels near the lower left corners of protein boxes. The following symbols are used to denote locations: E, extracellular; M, membrane anchored; and C, cytosol. Locations that can be inferred are not indicated. Protein boxes are organized in layers, which are indicated by shading. Stimulating antibodies are represented in the top layer, TCR/CD3 and CD28 are represented in the next layer, their direct interaction partners are indicated in the next layer, and so on. Elements of this map directly related to elements of the underlying rule-based model that it visualizes. A rule-based model is composed of molecule type definitions and rules, as well as specifications of rate laws, parameters, and initial conditions. Molecule type definitions of the model are illustrated here by protein boxes. Rules are illustrated by arrows. Each arrow corresponds to a single rule or a set of related rules. Numbers next to arrows reference rules presented in File A in File S1. **Figure S5 in File S2**. Principal component analysis of time-course data used to guide model specification and estimate model parameter values. Experimental time courses for the 16 pTyr sites included in the model were analyzed by principal component analysis and found to separate into three classes, which are distinguished by different background colors and labeled 1–3. Time courses in Class 1 correspond to pTyr sites that were observed to undergo increases in phosphorylation; according to the model, these increases occur through mechanisms that do not require prior ITAM phosphorylation. An example of a pTyr site in Class 1 is CD3G Y171, which in the model can be phosphorylated by LCK bound to CD28 through a constitutive interaction that does not require ITAM phosphorylation. Time courses in Class 2 correspond to pTyr sites that were also observed to undergo increases in phosphorylation; however, according to the model, these increases in phosphorylation occur through mechanisms that require ITAM phosphorylation. For example, ZAP70 must be recruited to a phosphorylated ITAM before it can be phosphorylated by LCK at Y493. Time courses in Class 3 correspond to pTyr sites were observed to undergo decreases in phosphorylation. **Figure S6 in File S2**. Experimental and simulated time courses. (**A** through **P**) Phosphorylation dynamics of the 16 pTyr sites used to guide model construction and estimate model parameters are plotted. Points represent the average of measurements from three independent phosphoproteomic experiments, with error bars representing standard deviations. Simulation results are plotted as solid lines. Experimental measurements and simulation results are normalized to baseline and log_2_ transformed. (**Q** and **R**) Measured phosphorylation dynamics of pTyr sites in PAG1, additional to the site shown in Panel m. The dynamics of these sites are similar to the dynamics of PAG1 pY163; these sites are not explicitly considered in the model. Note that the results presented here were presented earlier in Figs. 2–4 in different form. **Figure S7 in File S2**. PTPN11 levels in normal cells and in cells depleted of PTPN6. Immunoblots of PTPN11 (SHP-2) in normal cells (WT) and in cells depleted of PTPN6 (SHP-1 KD). Blots are representative of the results from multiple (at least two) experiments. **Figure S8 in File S2**. In vitro phosphatase activity of PTPN6. Immunoprecipitated LCK was treated or untreated with recombinant PTPN6 and immunoblotted using phospho-tyrosine specific antibodies as indicated. LCK specific antibodies were used to show equal amounts of immunoprecipitated LCK. Blots are representative of the results from multiple (at least two) experiments. **Figure S9 in File S2**. Disengagement of the shortcut pathway and engagement of the longer LCP2-dependent pathway to WAS activation. (**A**) Predicted association of NCK1/2 with CD3E at 0 and60 s of stimulation. The *y*-axis indicates the number of NCK molecules associated with TCR/CD3 complexes per cell. (**B**) Predicted association of NCK1/2 with phosphorylated LCP2 at 0 and 60 s of stimulation. The *y*-axis indicates the number of NCK molecules associated with LCP2 per cell. (**C**) Immunoblot of LCP2 phosphorylation at Y145 in normal (WT) and LCP2 KD cells stimulated for the indicated times. (**D**) Immunoblot of LCP2 phosphorylation at Y113 in normal (WT) and LCP2 KD cells stimulated for the indicated times. Blots are representative of the results from multiple (at least two) experiments. **Table S3 in File S2**. Proteins and pTyr sites included in the model for TCR signaling. **Table S4 in File S2**. Summary of earlier phosphoproteomic studies of TCR signaling. **Table S5 in File S2**. Comparison of selected models for immunoreceptor signaling in which phosphosite dynamics were considered.
(PDF)

File S3 **This PDF file provides the Supplementary Text.** **Supplementary Text S1 in File S3**. This PDF file provides extensive annotation of the model.
(PDF)
