# Supplementary material for: Phosphorylation Site Dynamics of Early T-cell Receptor Signaling
Source: PLoS One. 2014 Aug 22;9(8):e104240. doi: 10.1371/journal.pone.0104240 (PMC4141737; doi:10.1371/journal.pone.0104240)
Supplement: File S2 — This PDF file combines Figures S1–S9 and Tables S3–S5. Figure S1 in File S2. Overview of methodology and summary of main results. (A) An integrated experimental and model-based approach was used to characterize initial phosphorylation events in TCR signaling, generate non-trivial predictions, and test these predictions. A model based solely on previously elucidated mechanisms of TCR signaling did not reproduce the phosphorylation dynamics observed for the following five sites: LCK Y192, DOK1 Y449, DOK2 Y299, PAG1 Y417, and WAS Y291. Incorporation of novel mechanisms enabled the dynamics of these sites to be reproduced and led to generation of predictions that were tested experimentally. (B) Proposed roles of PTPN6 in early and late signaling. In early signaling (bold lines), PTPN6 plays a positive role by dephosphorylating negative regulatory sites, including LCK Y192, PAG1 Y163, DOK1 Y449, and DOK2 Y299. Later in signaling (thin lines), the negative regulatory capabilities of PTPN6 become dominant. (C) Proposed dual pathways for activation of WAS. In early signaling (bold lines), WAS is recruited to the plasma membrane through interaction with NCK1/2 in association with CD3E. As signaling progresses over time, the longer pathway for WAS recruitment (thin lines), which is dependent on LCP2, becomes dominant. (D) A PTPN6-mediated positive feedback loop in which PTPN6 dephosphorylates LCK Y192, thereby enhancing the ability of the LCK SH2 domain to interact with pTyr sites. LCK activates PTPN6 through phosphorylation and direct interaction. (E) In a second PTPN6-mediated positive feedback loop, LCK phosphorylates and activates PTPN6. PTPN6 dephosphorylates PAG1, which reduces the ability of PAG1 to co-localize LCK and CSK, which reduces phosphorylation of LCK at its inhibitory C-terminal tyrosine and relieves autoinhibition. Figure S2 in File S2. Enrichment analysis. For proteins containing regulated pTyr sites, we tested for enrichment of associated GO terms compared [file pone.0104240.s002.pdf]

Supplementary File S2: Figures S1–S9 and Tables S3–S5

Phosphorylation site dynamics of early T-cell receptor signaling

L. A. Chylek, V. Akimov, J. Dengjel, K. T. G. Rigbolt, B. Hu, W. S. Hlavacek, B. Blagoev

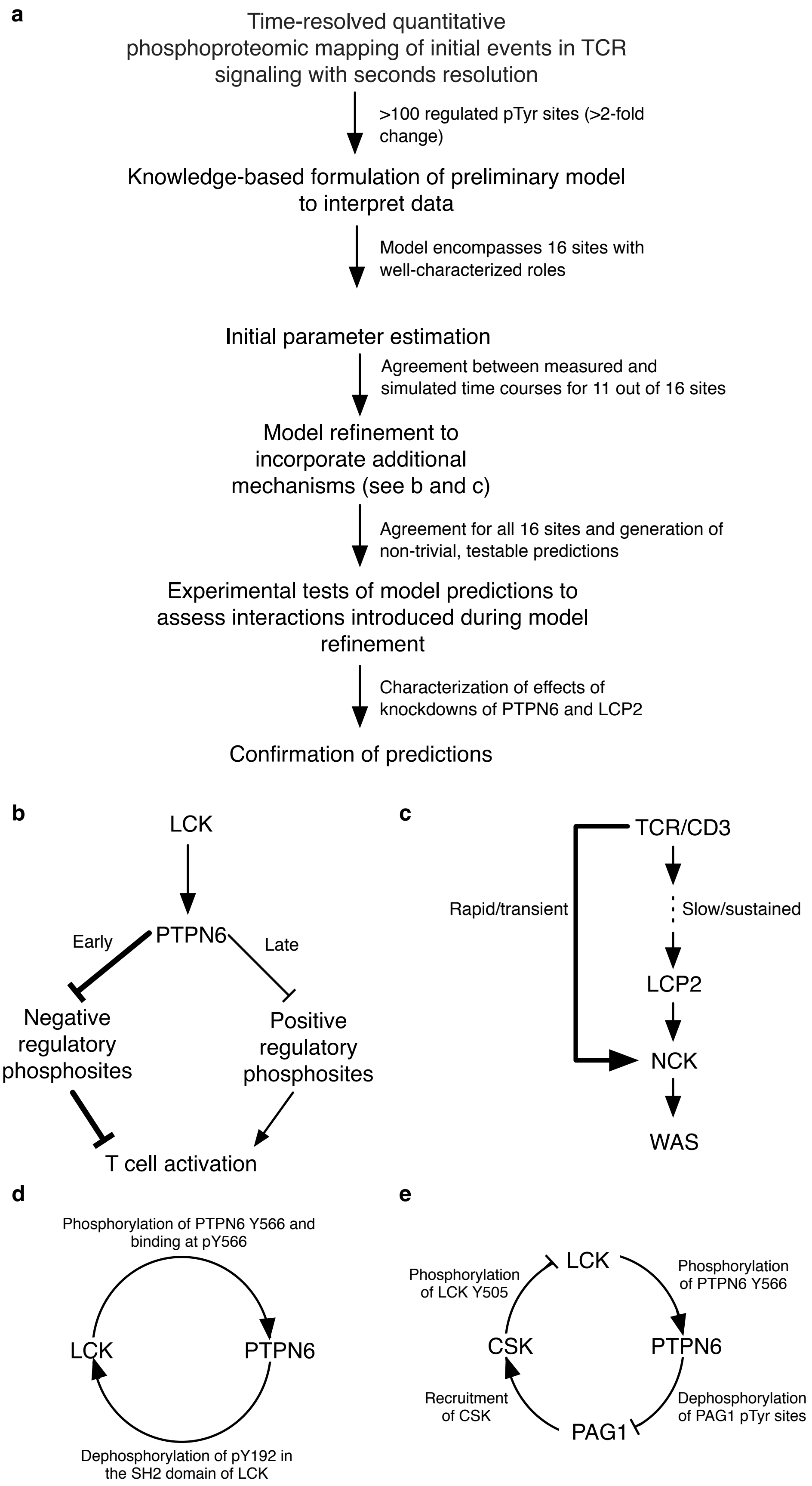
a
Time-resolved quantitative phosphoproteomic mapping of initial events in TCR signaling with seconds resolution
>100 regulated pTyr sites (>2-fold change)
Knowledge-based formulation of preliminary model to interpret data
Model encompasses 16 sites with well-characterized roles
Initial parameter estimation
Agreement between measured and simulated time courses for 11 out of 16 sites
Model refinement to incorporate additional mechanisms (see b and c)
Agreement for all 16 sites and generation of non-trivial, testable predictions
Experimental tests of model predictions to assess interactions introduced during model refinement
Characterization of effects of knockdowns of PTPN6 and LCP2
Confirmation of predictions
b
LCK
PTPN6
Early
Late
Negative regulatory phosphosites
Positive regulatory phosphosites
T cell activation
c
TCR/CD3
Rapid/transient
Slow/sustained
LCP2
NCK
WAS
d
Phosphorylation of PTPN6 Y566 and binding at pY566
LCK
PTPN6
Dephosphorylation of pY192 in the SH2 domain of LCK
e
Phosphorylation of LCK Y505
LCK
Phosphorylation of PTPN6 Y566
CSK
PTPN6
Recruitment of CSK
PAG1
Dephosphorylation of PAG1 pTyr sites

Figure S1. Overview of methodology and summary of main results. (A) An integrated experimental and model-based approach was used to characterize initial phosphorylation events in TCR signaling, generate non-trivial predictions, and test these predictions. A model based solely on previously elucidated mechanisms of TCR signaling did not reproduce the phosphorylation dynamics observed for the following five sites: LCK Y192, DOK1 Y449, DOK2 Y299, PAG1 Y417, and WAS Y291. Incorporation of novel mechanisms enabled the dynamics of these sites to be reproduced and led to generation of predictions that were tested experimentally. **(B)** Proposed roles of PTPN6 in early and late signaling. In early signaling (bold lines), PTPN6 plays a positive role by dephosphorylating negative regulatory sites, including LCK Y192, PAG1 Y163, DOK1 Y449, and DOK2 Y299. Later in signaling (thin lines), the negative regulatory capabilities of PTPN6 become dominant. **(C)** Proposed dual pathways for activation of WAS. In early signaling (bold lines), WAS is recruited to the plasma membrane through interaction with NCK1/2 in association with CD3E. As signaling progresses over time, the longer pathway for WAS recruitment (thin lines), which is dependent on LCP2, becomes dominant. **(D)** A PTPN6-mediated positive feedback loop in which PTPN6 dephosphorylates LCK Y192, thereby enhancing the ability of the LCK SH2 domain to interact with pTyr sites. LCK activates PTPN6 through phosphorylation and direct interaction. **(E)** In a second PTPN6-mediated positive feedback loop, LCK phosphorylates and activates PTPN6. PTPN6 dephosphorylates PAG1, which reduces the ability of PAG1 to co-localize LCK and CSK, which reduces phosphorylation of LCK at its inhibitory C-terminal tyrosine and relieves autoinhibition.

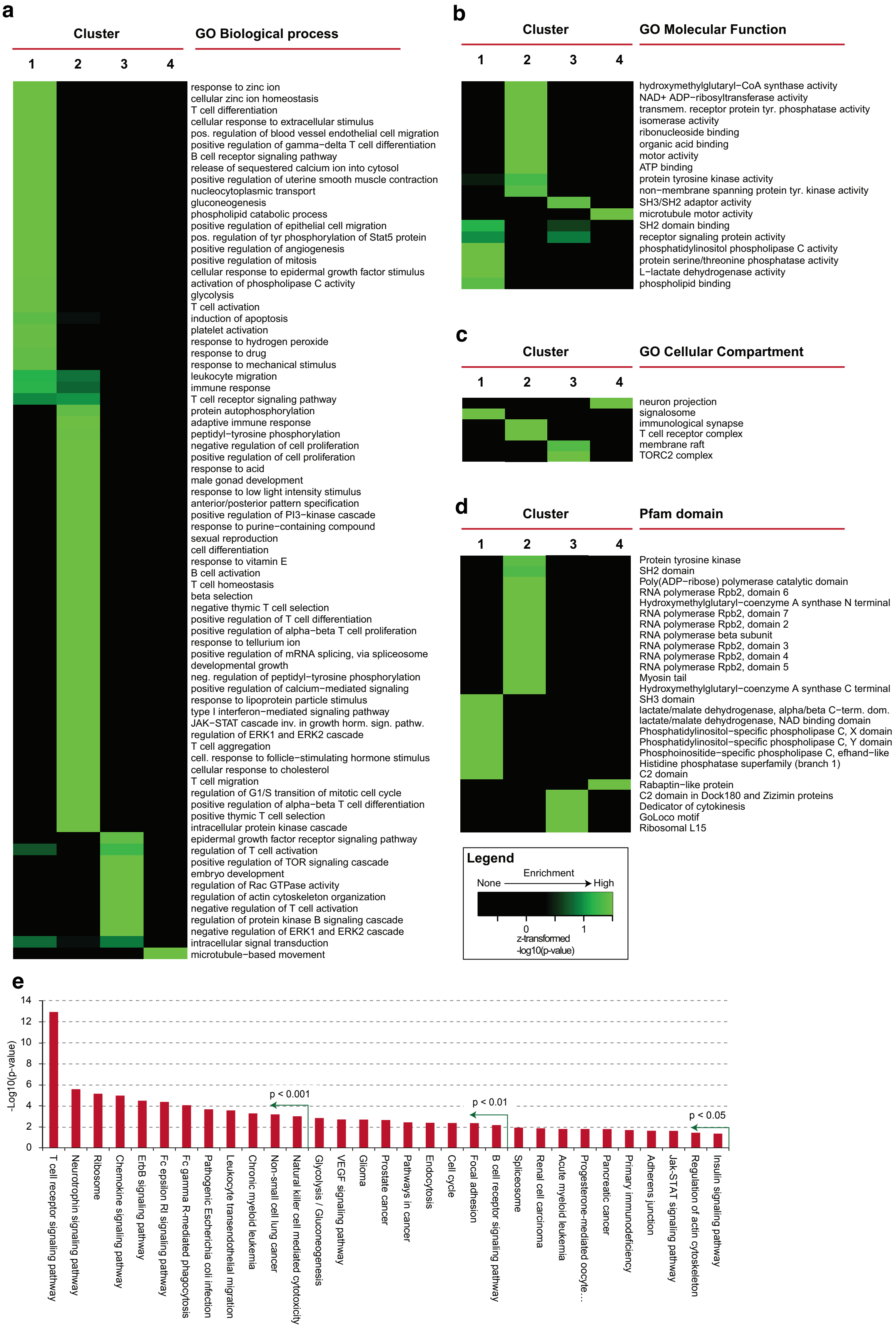

a
Cluster
GO Biological process
1
2
3
4
response to zinc ion
cellular zinc ion homeostasis
T cell differentiation
cellular response to extracellular stimulus
pos. regulation of blood vessel endothelial cell migration
positive regulation of gamma−delta T cell differentiation
B cell receptor signaling pathway
release of sequestered calcium ion into cytosol
positive regulation of uterine smooth muscle contraction
nucleocytoplasmic transport
gluconeogenesis
phospholipid catabolic process
positive regulation of epithelial cell migration
pos. regulation of tyr phosphorylation of Stat5 protein
positive regulation of angiogenesis
positive regulation of mitosis
cellular response to epidermal growth factor stimulus
activation of phospholipase C activity
glycolysis
T cell activation
induction of apoptosis
platelet activation
response to hydrogen peroxide
response to drug
response to mechanical stimulus
leukocyte migration
immune response
T cell receptor signaling pathway
protein autophosphorylation
adaptive immune response
peptidyl−tyrosine phosphorylation
negative regulation of cell proliferation
positive regulation of cell proliferation
response to acid
male gonad development
response to low light intensity stimulus
anterior/posterior pattern specification
positive regulation of PI3−kinase cascade
response to purine−containing compound
sexual reproduction
cell differentiation
response to vitamin E
B cell activation
T cell homeostasis
beta selection
negative thymic T cell selection
positive regulation of T cell differentiation
positive regulation of alpha−beta T cell proliferation
response to tellurium ion
positive regulation of mRNA splicing, via spliceosome
developmental growth
neg. regulation of peptidyl−tyrosine phosphorylation
positive regulation of calcium−mediated signaling
response to lipoprotein particle stimulus
type I interferon−mediated signaling pathway
JAK−STAT cascade inv. in growth horm. sign. pathw.
regulation of ERK1 and ERK2 cascade
T cell aggregation
cell. response to follicle−stimulating hormone stimulus
cellular response to cholesterol
T cell migration
regulation of G1/S transition of mitotic cell cycle
positive regulation of alpha−beta T cell differentiation
positive thymic T cell selection
intracellular protein kinase cascade
epidermal growth factor receptor signaling pathway
regulation of T cell activation
positive regulation of TOR signaling cascade
embryo development
regulation of Rac GTPase activity
regulation of actin cytoskeleton organization
negative regulation of T cell activation
regulation of protein kinase B signaling cascade
negative regulation of ERK1 and ERK2 cascade
intracellular signal transduction
microtubule−based movement
b
Cluster
GO Molecular Function
1
2
3
4
hydroxymethylglutaryl−CoA synthase activity
NAD+ ADP−ribosyltransferase activity
transmem. receptor protein tyr. phosphatase activity
isomerase activity
ribonucleoside binding
organic acid binding
motor activity
ATP binding
protein tyrosine kinase activity
non−membrane spanning protein tyr. kinase activity
SH3/SH2 adaptor activity
microtubule motor activity
SH2 domain binding
receptor signaling protein activity
phosphatidylinositol phospholipase C activity
protein serine/threonine phosphatase activity
L−lactate dehydrogenase activity
phospholipid binding
c
Cluster
GO Cellular Compartment
1
2
3
4
neuron projection
signalosome
immunological synapse
T cell receptor complex
membrane raft
TORC2 complex
d
Cluster
Pfam domain
1
2
3
4
Protein tyrosine kinase
SH2 domain
Poly(ADP−ribose) polymerase catalytic domain
RNA polymerase Rpb2, domain 6
Hydroxymethylglutaryl−coenzyme A synthase N terminal
RNA polymerase Rpb2, domain 7
RNA polymerase Rpb2, domain 2
RNA polymerase beta subunit
RNA polymerase Rpb2, domain 3
RNA polymerase Rpb2, domain 4
RNA polymerase Rpb2, domain 5
Myosin tail
Hydroxymethylglutaryl−coenzyme A synthase C terminal
SH3 domain
lactate/malate dehydrogenase, alpha/beta C−term. dom.
lactate/malate dehydrogenase, NAD binding domain
Phosphatidylinositol−specific phospholipase C, X domain
Phosphatidylinositol−specific phospholipase C, Y domain
Phosphoinositide−specific phospholipase C, efhand−like
Histidine phosphatase superfamily (branch 1)
C2 domain
Rabaptin−like protein
C2 domain in Dock180 and Zizimin proteins
Dedicator of cytokinesis
GoLoco motif
Ribosomal L15
Legend
Enrichment
None
High
0
1
z-transformed
-log10(p-value)
e
-Log10(p-value)
14
12
10
8
6
4
2
0
p < 0.001
p < 0.01
p < 0.05
T cell receptor signaling pathway
Neurotrophin signaling pathway
Ribosome
Chemokine signaling pathway
ErbB signaling pathway
Fc epsilon RI signaling pathway
Fc gamma R-mediated phagocytosis
Pathogenic Escherichia coli infection
Leukocyte transendothelial migration
Chronic myeloid leukemia
Non-small cell lung cancer
Natural killer cell mediated cytotoxicity
Glycolysis / Gluconeogenesis
VEGF signaling pathway
Glioma
Prostate cancer
Pathways in cancer
Endocytosis
Cell cycle
Focal adhesion
B cell receptor signaling pathway
Spliceosome
Renal cell carcinoma
Acute myeloid leukemia
Progesterone-mediated oocyte...
Pancreatic cancer
Primary immunodeficiency
Adherens junction
Jak-STAT signaling pathway
Regulation of actin cytoskeleton
Insulin signaling pathway

Figure S2. Enrichment analysis. For proteins containing regulated pTyr sites, we tested for enrichment of associated GO terms compared to proteins containing detected but unregulated pTyr sites (i.e., pTyr sites for which phosphorylation changed less than two-fold). **(A)** Cluster-specific enrichment analysis based on "biological process" terms. **(B)** Cluster-specific enrichment analysis based on "molecular function" terms. **(C)** Cluster-specific enrichment analysis based on "cellular compartment" terms. **(D)** We also tested for enrichment of associated Pfam domain names. In A through D, color is used to indicate the negative logarithm (base 10) of the *z*-transformed *p*-value associated with each term. The lighest shade of green corresponds to the highest level of enrichment. Black corresponds to no enrichment. **(E)** Information about detected pTyr sites was uploaded to the DAVID resource and processed using default parameters to identify pathways enriched for regulated pTyr sites. The *y*-axis reports the negative logarithm (base 10) of the *p*-value for each of the indicated pathways. Pathway enrichment scores are reported on the *y*-axis. The most enriched pathway is the "T cell receptor signaling pathway."

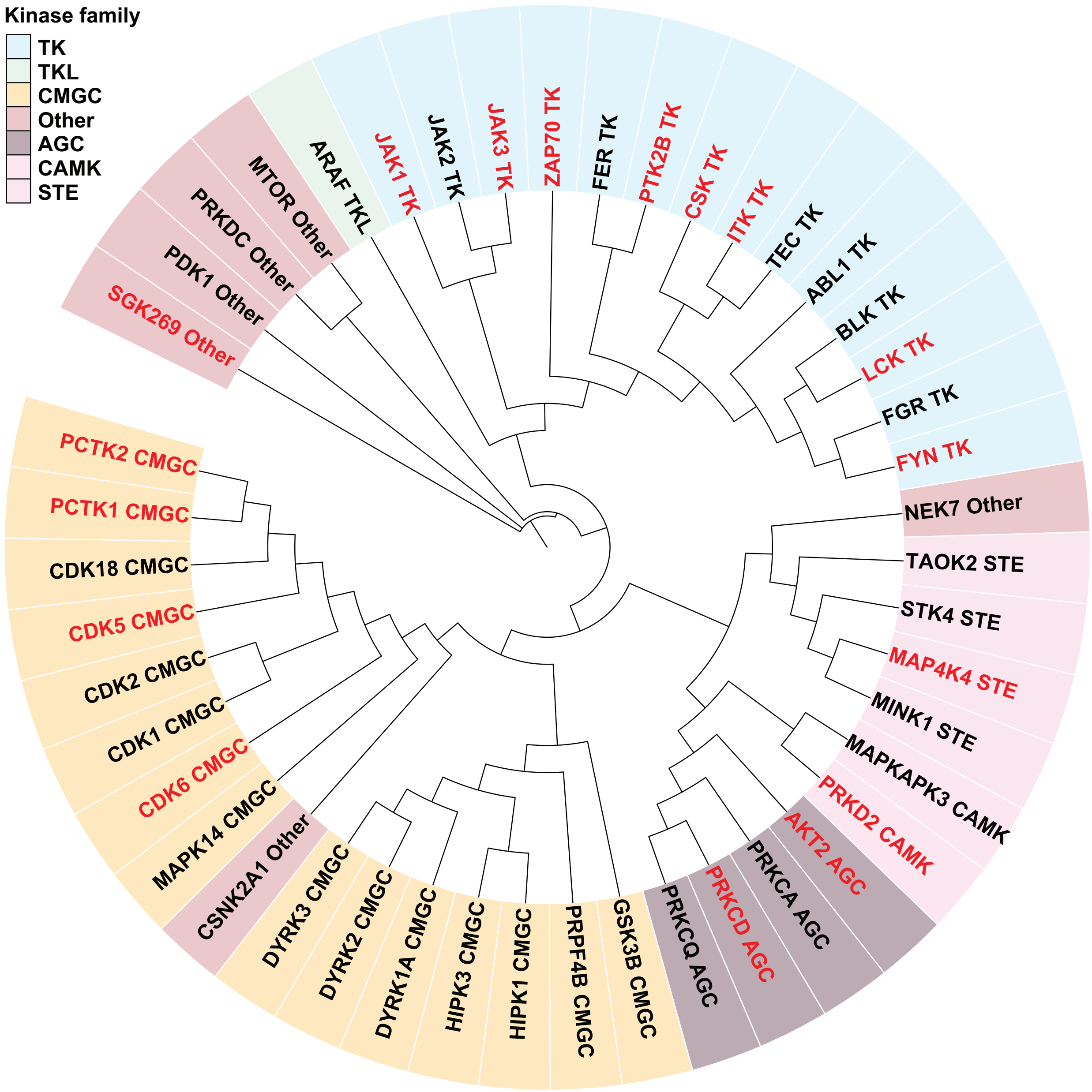

Figure S3. Phylogenetic relationships of protein kinases with detected pTyr sites. The tree shown was built based on kinase domain sequences. Protein kinases that contain regulated pTyr sites are indicated with red lettering; these kinases are also represented in Fig. 1h. Kinase families are indicated by background colors. The following abbreviations are used for protein kinase family names: TK, tyrosine kinases; TKL, tyrosine kinase-like; CMGC, the CDK/MAPK/GSK3/CLK group; AGC, protein kinase A, G, and C families; CAMK, calcium and calmodulin regulated kinases; and STE, homologs of the yeast STE7, STE11, and STE20 genes.

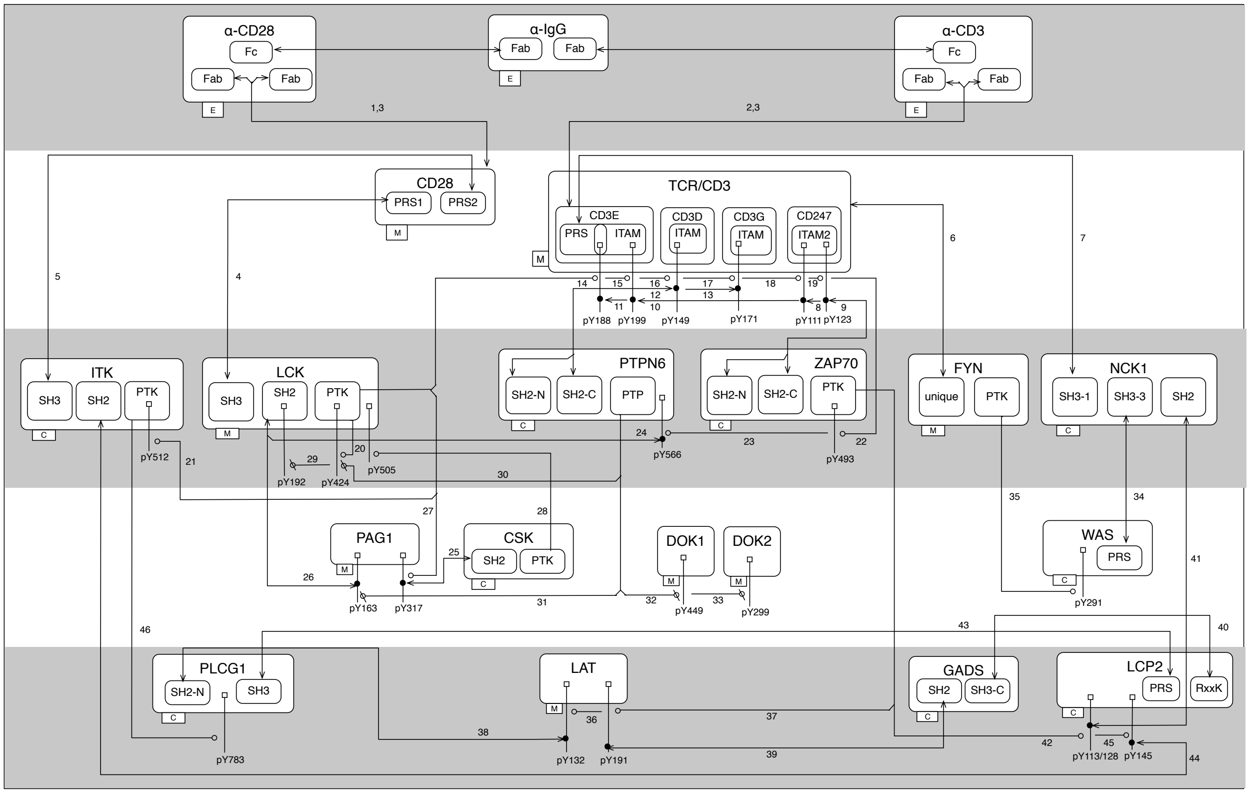

Figure S4. Visualization of model. Proteins, domains, and linear motifs are represented as boxes, which are nested to indicate structural relationships. Lines that begin and end with an arrowhead represent direct binding interactions. Arrowheads point to functional components that mediate protein-protein interactions. Lines that originate at a box representing an enzyme (a kinase or phosphatase) and end with an open circle, or open circle overlayed with a diagonal bar, indicate enzyme-substrate relationships. An open circle denotes phosphorylation and an open cirlce overlayed with a diagonal bar denotes dephosphorylation. Flags (vertical lines connected to a small square box at top and a text label at bottom) represent sites of post-translational modification. All of these sites are pTyr sites. Compartmental locations of proteins are indicated by boxed labels near the lower left corners of protein boxes. The following symbols are used to denote locations: E, extracellular; M, membrane anchored; and C, cytosol. Locations that can be inferred are not indicated. Protein boxes are organized in layers, which are indicated by shading. Stimulating antibodies are represented in the top layer, TCR/CD3 and CD28 are represented in the next layer, their direct interaction partners are indicated in the next layer, and so on. Elements of this map directly related to elements of the underlying rule-based model that it visualizes. A rule-based model is composed of molecule type definitions and rules, as well as specifications of rate laws, parameters, and initial conditions. Molecule type definitions of the model are illustrated here by protein boxes. Rules are illustrated by arrows. Each arrow corresponds to a single rule or a set of related rules. Numbers next to arrows reference rules presented in File S1.

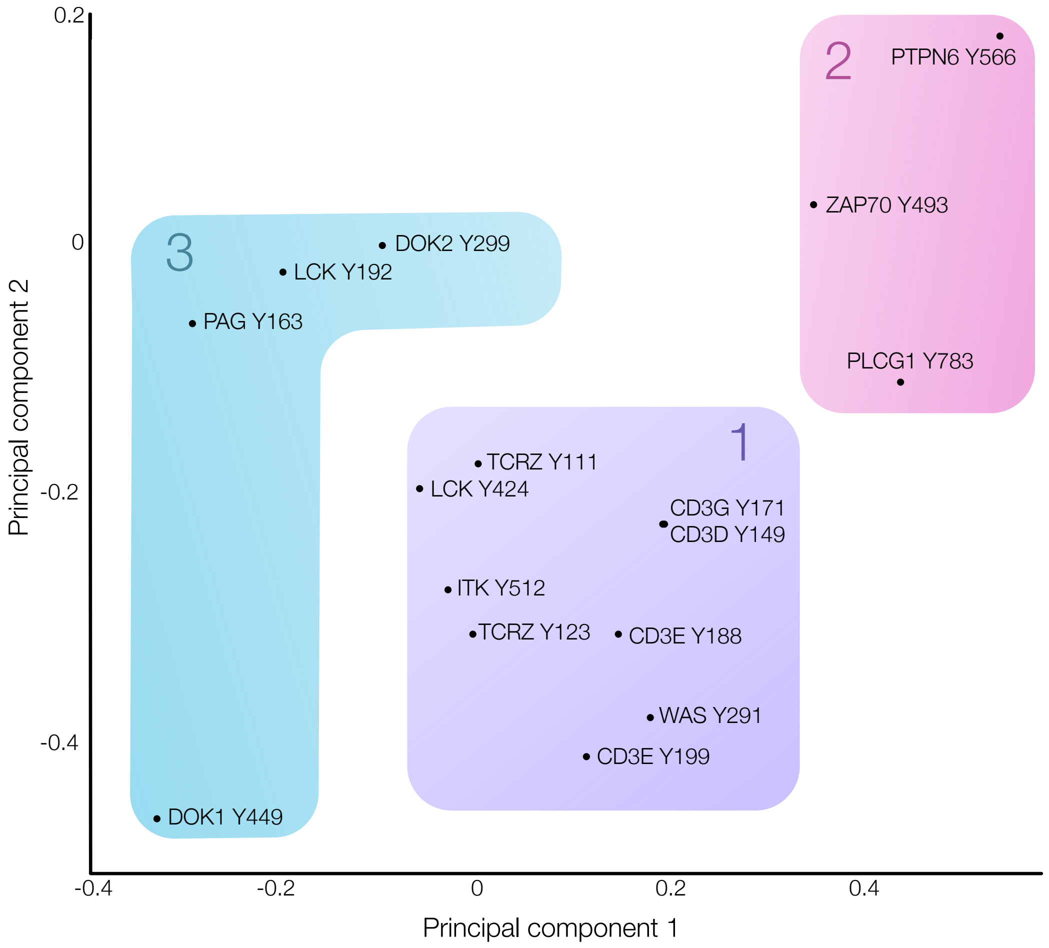

Figure S5. Principal component analysis of time-course data used to guide model specification and estimate model parameter values. Experimental time courses for the 16 pTyr sites included in the model were analyzed by principal component analysis and found to separate into three classes, which are distinguished by different background colors and labeled 1–3. Time courses in Class 1 correspond to pTyr sites that were observed to undergo increases in phosphorylation; according to the model, these increases occur through mechanisms that do not require prior ITAM phosphorylation. An example of a pTyr site in Class 1 is CD3G Y171, which in the model can be phosphorylated by LCK bound to CD28 through a constitutive interaction that does not require ITAM phosphorylation. Time courses in Class 2 correspond to pTyr sites that were also observed to undergo increases in phosphorylation; however, according to the model, these increases in phosphorylation occur through mechanisms that require ITAM phosphorylation. For example, ZAP70 must be recruited to a phosphorylated ITAM before it can be phosphorylated by LCK at Y493. Time courses in Class 3 correspond to pTyr sites were observed to undergo decreases in phosphorylation.

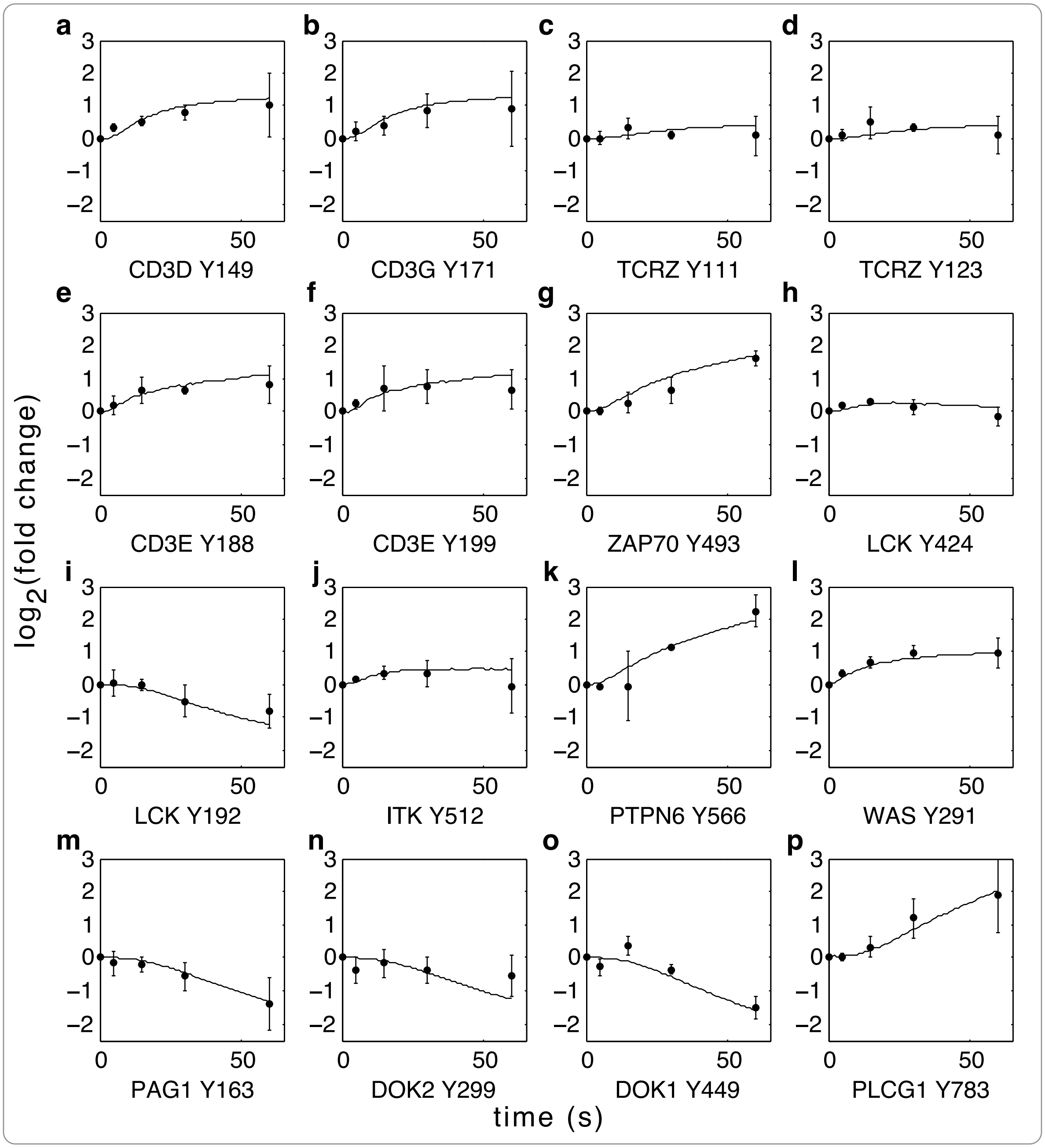

log2(fold change)
a CD3D Y149
b CD3G Y171
c TCRZ Y111
d TCRZ Y123
e CD3E Y188
f CD3E Y199
g ZAP70 Y493
h LCK Y424
i LCK Y192
j ITK Y512
k PTPN6 Y566
l WAS Y291
m PAG1 Y163
n DOK2 Y299
o DOK1 Y449
p PLCG1 Y783
time (s)

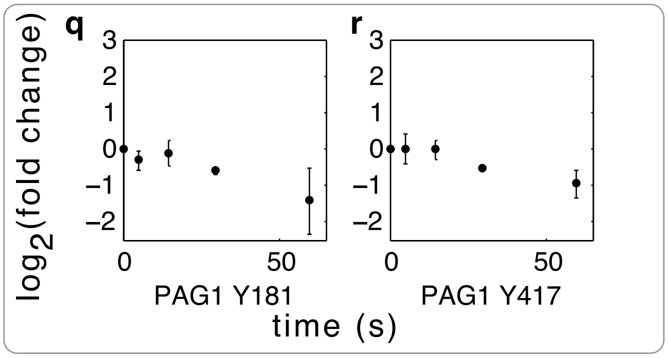

log2(fold change)
q PAG1 Y181
r PAG1 Y417
time (s)

Figure S6. Experimental and simulated time courses. (**A** through **P**) Phosphorylation dynamics of the 16 pTyr sites used to guide model construction and estimate model parameters are plotted. Points represent the average of measurements from three independent phosphoproteomic experiments, with error bars representing standard deviations. Simulation results are plotted as solid lines. Experimental measurements and simulation results are normalized to baseline and $\log_2$ transformed. **(Q** and **R)** Measured phosphorylation dynamics of pTyr sites in PAG1, additional to the site shown in Panel m. The dynamics of these sites are similar to the dynamics of PAG1 pY163; these sites are not explicitly considered in the model. Note that the results presented here were presented earlier in Figs. 2–4 in different form.

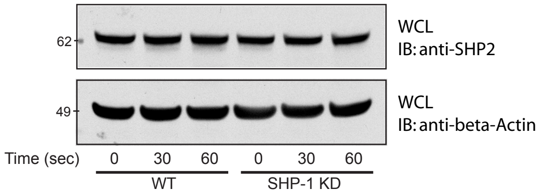

Figure S7. PTPN11 levels in normal cells and cells depleted of PTPN6. Immunoblots of PTPN11 (SHP-2) in normal cells (WT) and in cells depleted of PTPN6 (SHP-1 KD). Blots are representative of the results from multiple (at least two) experiments.

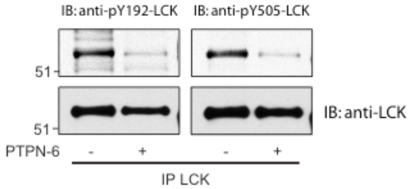

Figure S8. In vitro phosphatase activity of PTPN6. Immunoprecipitated LCK was treated or untreated with recombinant PTPN6 and immunoblotted using phospho-tyrosine specific antibodies as indicated. LCK specific antibodies were used to show equal amounts of immunoprecipitated LCK. Blots are representative of the results from multiple (at least two) experiments.

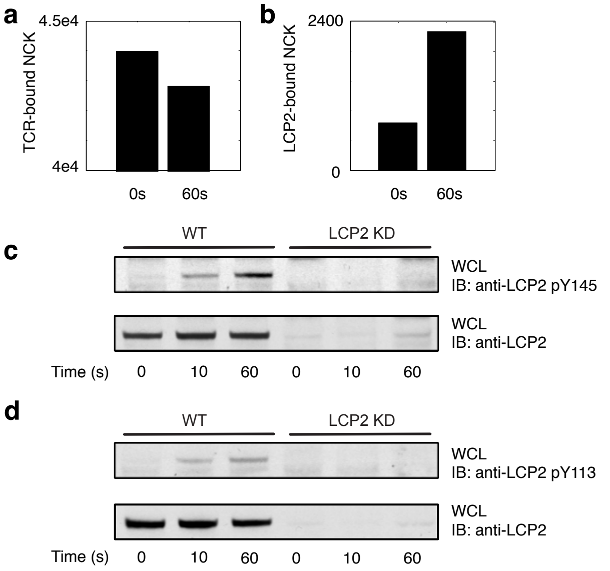

Figure S9. **Disengagement of the shortcut pathway and engagement of the longer LCP2-dependent pathway to WAS activation**. **(A)** Predicted association of NCK1/2 with CD3E at 0 and 60 s of stimulation. The *y*-axis indicates the number of NCK molecules associated with TCR/CD3 complexes per cell. **(B)** Predicted association of NCK1/2 with phosphorylated LCP2 at 0 and 60 s of stimulation. The *y*-axis indicates the number of NCK molecules associated with LCP2 per cell. **(C)** Immunoblot of LCP2 phosphorylation at Y145 in normal (WT) and LCP2 KD cells stimulated for the indicated times. **(D)** Immunoblot of LCP2 phosphorylation at Y113 in normal (WT) and LCP2 KD cells stimulated for the indicated times. Blots are representative of the results from multiple (at least two) experiments.

Table S3. Proteins and pTyr sites included in the model for TCR signaling.

Protein	Tyrosine residue number	Corresponding row in Supplementary Table 1	Comment
CD247 (TCR ζ chain)	111	62	N-terminal tyrosine in second ζ ITAM
CD247 (TCR ζ chain)	123	63	C-terminal tyrosine in second ζ ITAM
CD3E	188	70	Shared tyrosine of the CD3ε PRS and ITAM
CD3E	199	71	C-terminal tyrosine in CD3ε ITAM
CD3D	149	68	N-terminal tyrosine in CD3δ ITAM
CD3G	171	73	C-terminal tyrosine in CD3γ ITAM
LCK	192 [a]	281	Located in the LCK SH2 domain
LCK	394	278	Activation loop
LCK	505 [a,b]	n/a; see Fig. 3 for immunoblot data	Phosphorylation of this site promotes the autoinhibited conformation of LCK
ZAP70	493	664	Activation loop
PTPN6 (SHP-1)	566	452	Phosphorylation of this site is associated with activation, and is also a binding site for SH2 domains
ITK	512	252	Activation loop
PAG1	163 [a]	364	Binding site for SRC-family kinases (SFKs)
PAG1	317 [a]	n/a	CSK binding site
DOK1	449 [a]	145	CSK binding site
DOK2	299 [a]	146	RASA1 (p120 RasGAP) binding site
WAS	291	646	Phosphorylation stabilizes the active conformation of WAS
PLCG1 (PLC□1)	783	407	Phosphorylation of this site activates phospholipase activity
NCK1/2	n/a [c]	n/a	Adaptor protein
LAT	132 [b]	n/a	PLCγ1 binding site
LAT	191 [b]	n/a	GRAP2 (Gads) binding site
LCP2 (SLP-76)	113/128 [b]	n/a	NCK1/2 binding sites
FYN	n/a [d]	n/a	SFK
CSK	n/a [c]	n/a	Phosphorylates C-terminal regulatory tyrosines of SFKs, promoting SFK autoinhibition
GRAP2 (Gads)	n/a [c]	n/a	Adaptor protein

[a] Phosphorylation of this site is associated with negative regulation.
[b] No peptide corresponding to this site was detected, but this site is known to be involved in TCR signaling.
[c] This protein is known to be involved in TCR signaling, but its phosphorylation is not required for the interactions considered in our model.
[d] Phosphorylation of a peptide corresponding to the activation loop of FYN was detected: LIEDNEY(ph)TAR. This sequence is shared by multiple SFKs. For simplicity, phosphorylation of FYN is not included in the model.

Table S4. Summary of earlier phosphoproteomic studies of TCR signaling.

Study	Time points (sec) [a]	Stimulus	Conditions
A [73]	0, 120, 180, 300, 420, 600	Anti-CD3, anti-CD4, secondary antibody	ZAP70 null and reconstituted Jurkat T cells
B [35]	0, 60, 90, 120, 180, 300, 600	Anti-CD3, anti-CD4, secondary antibody	LCP2 (SLP-76) deficient and reconstituted Jurkat T cells
C [74]	0, 30, 120, 300, 600	Anti-CD3	LCP2 deficient and WT Jurkat T cells
D [75]	0, 300, 900, 3600	Anti-CD3, secondary antibody	Jurkat T cells
E [76]	0, 300	Anti-CD3 and anti-CD28, with and without secondary antibody	Jurkat T cells
F [77]	0, 300	Anti-CD3 biotin and streptavidin	CD4+ T cells from diabetes-susceptible and diabetes-resistant mice
G [78]	0, 180	Anti-CD3	Jurkat T cells
H [79]	0, 300	Anti-CD3	Primary human T cells
I (this work)	0, 5, 15, 30, 60	Anti-CD3, anti-CD28, and secondary antibody	Jurkat T cells

[a] In some cases, the measurement indicated in this table as that taken at time $t = 0$ is described in the original report as a control experiment (no stimulation).

Table S5. Comparison of selected models for immunoreceptor signaling in which phosphosite dynamics were considered.

Model[a]	Specific sites captured in model	Lumped sites captured in model[b]	Experimental time courses generated/used to identify parameter values	Time points considered in experiments (s)	Total number of data points	Method used to measure phosphosite dynamics
A1 [80]	1	3	3[c]	0, 85, 210, 505, 900, 1920, 4200	21	Western blot
A2 [81]	0	1	2[c]	0, 120, 240, 360, 960, 1800, 3780	14	Western blot
B1 [18]	5	5	0[e]	n/a	n/a	n/a
B2 [19]	1	1	0[e]	n/a	n/a	n/a
C1 [82, 83]	2[d]	5[d]	0[e]	n/a	n/a	n/a
C2 [84]	0	1	0[e]	n/a	n/a	n/a
C3 [85]	2	8	4[c]	0, 120, 600, 1200, 3600	20	Western blot
C4 [20]	0	3	0[e]	n/a	n/a	n/a
C5 (this work)	21	1	16	0, 5, 15, 30, 60	80	Mass spectrometry

[a]Models are labeled to reflect the system of study. "A" refers to Fc□RI signaling, "B" refers to B cell antigen receptor signaling, and "C" refers to T cell receptor signaling.

[b]A variety of site lumping schemes have been used by modelers. For example, in the study of Faeder et al. [80], multiple tyrosine residues in the linker region of Syk were lumped together as one site with two states (unphosphorylated and phosphorylated). In Mukherjee et al. [19], four tyrosine residues in two ITAMs of the B cell antigen receptor were lumped together as one site with four states (unphosphorylated, partially phosphorylated, one ITAM fully phosphorylated, and both ITAMs fully phosphorylated).

[c]Measurements of phosphorylation were performed without site-specific resolution, and may thus reflect contributions of multiple phosphosites within a protein.

[d]Number reflects the simpler model of Lipniacki et al. [83].

[e]Phosphorylation time courses were not measured in this study.
