# Supplementary material for: Phosphorylation Site Dynamics of Early T-cell Receptor Signaling
Source: PLoS One. 2014 Aug 22;9(8):e104240. doi: 10.1371/journal.pone.0104240 (PMC4141737; doi:10.1371/journal.pone.0104240)
Supplement: File S3 — This PDF file provides the Supplementary Text. Supplementary Text S1 in File S3. This PDF file provides extensive annotation of the model. (PDF) [file pone.0104240.s003.pdf]

Supplementary Text

Introduction

This supplementary text is meant to serve as a model guide [22] that annotates the model for T-cell receptor (TCR) signaling that was used for analysis of temporal phosphoproteomic data. The main parts of this document are:

1. A list of abbreviations used frequently in the model and in this discussion,
2. An introduction to the modeling method and syntax,
3. A summary of information about molecules and their encodings in the model, and
4. Annotated encodings of rules representing biomolecular interactions in the model.

An executable model specification is given in Supplementary File S1, which can be simulated using the protocol of Supplementary File S2. Table S2 is a spreadsheet of parameter estimates used in the model. The model is visualized in Fig. 2, and in greater detail in Supplementary Fig. S4.

Frequently used abbreviations

- ITAM = immunoreceptor tyrosine-based activation motif
- PRS = proline-rich sequence
- PTK = protein tyrosine kinase
- PTP = protein tyrosine phosphatase
- SFK = Src-family kinase
- SH2 = Src homology 2
- SH3 = Src homology 3
- pY = phosphorylated tyrosine
- Y = tyrosine

Modeling method

To match the level of detail present in the data (population-averaged phosphorylation levels of individual amino acid residues), reactions must factor in specific protein sites, which increases the number of species that must be considered. For example, if one considers three sites of phosphorylation in the kinase LCK (Y192, Y394, and Y505, all of which are considered in our model), the molecule has $2^3 = 8$ possible phosphorylation states. When the multiple binding and phosphorylation states of all proteins of interest are considered, the large number of possible species is an obstacle to formulation of a model via traditional means, such as coupled ordinary differential equations. An alternative means of model specification that captures chemical kinetics like a traditional model but is better suited for modeling with site-specific resolution is rule-based modeling. In this approach, a domain-specific language is used to define local rules for biomolecular interactions. The modeling language that we have used is the BioNetGen language

(BNGL) [65], which is closely related to Kappa (http://www.kappalanguage.org/). BNGL and Kappa are the most widely used languages for specification of rule-based models. There is currently no universal standard for encoding of rule-based models, although the SBML multi package (http://sbml.org/Documents/Specifications/SBML_Level_3/Packages/multi) is currently in development and is intended to fill this role.

A number of reviews and introductory guides to rule-based modeling are available [14, 15] [86, 87, 88, 89, 90]. The two main elements of a rule-based model that are discussed in this guide are molecule type definitions and the rules themselves. A molecule type definition gives the protein's name, its components, and the possible internal states of those components (if applicable). For example, the molecule type definition `ZAP70(SH2,Y493~0~P)` states that the protein ZAP70 has two components: SH2 and Y493. Furthermore, Y493 has two possible internal states: unphosphorylated (0) and phosphorylated (P).

Rules are used to specify transformations that affect components of molecules, such as formation of a bond or a post-translational modification (represented as a change of a component's internal state). An example of a rule follows:

```
ZAP70(SH2) + TCR(Y111~P) <-> ZAP70(SH2!1).TCR(Y111~P!1) kf1,kr1
```

This rule states that a bond (labeled "1" and prefixed by "!") can form between the SH2 domain in ZAP70 and phosphorylated Y111 in the TCR, forming a complex between the two molecules. Bonds between molecules are indicated by name sharing. A complex is indicated by the presence of a dot operator (".") on the right-hand side. It is assumed that Y493 of ZAP70 does not affect the interaction. (Recall the molecule type definition given above for ZAP70.) Thus, Y493 is omitted from the rule. However, if it is known that additional components influence an interaction (e.g., through allostery), they can be included to make a rule more specific. The reactions implied by the rule above proceed with the forward rate constant `kf1` and reverse rate constant `kr1`.

Proteins in Model

1. **Crosslinking reagents**
   Jurkat T cells were stimulated with anti-CD3, anti-CD28, and secondary antibodies, which are commonly used reagents for experimental stimulation of T cells [91] because antibody-mediated crosslinking recapitulates intracellular signaling events that lead to cellular activation [23].

   a. **α-CD28**
   α-CD28 antibody (CD28.2) was purchased from Santa Cruz Biotechnology, Inc. It is a mouse monoclonal antibody raised against human CD28-transfected murine T cell hybridoma.

   b. **α-CD3**
   α-CD3 antibody (HIT3a) was purchased from Santa Cruz Biotechnology, Inc. It is a mouse monoclonal antibody.

   c. **α-IgG**
   α-IgG antibody (Z 0420) was purchased from DakoCytomation. It is a polyclonal goat anti-mouse antibody.

   These antibodies trigger signaling through clustering of TCR and CD28 molecules, which mimics the outcome of interactions between a T cell and an antigen-presenting cell. These antibodies have

the potential to induce large receptor/co-receptor oligomers, but as a simplification that captures the essential action of these stimuli, we model them as three virtual ligands that induce CD28 homodimers (Ligand 1), CD28/TCR heterodimers (Ligand 2), and TCR homodimers (Ligand 3).

Molecule type definitions:

a. `Lig1(aCD28,aCD28)`

b. `Lig2(aCD28,aCD3)`

c. `Lig3(aCD3,aCD3)`

The components `aCD3` and `aCD28` are considered to be binding sites that interact with CD3 and CD28, respectively.

2. **TCR/CD3 complex.**
TCR/CD3 is a multimeric complex of $\alpha\beta$, $\gamma\epsilon$, and $\delta\epsilon$ heterodimers and a $\zeta\zeta$ homodimer [92, 93, 94]. The CD3 ϵ, γ, and δ molecules each contain one immunoreceptor tyrosine-based activation motif (ITAM), and ζ contains three. These motifs comprise two tyrosines on either side of a consensus sequence [95]. Phosphorylation of a ζ ITAM allows the TCR to recruit the kinase ZAP70 [96]. The extracellular portion of CD3ϵ is recognized by α-CD3 antibodies [97, 98, 99]. The ITAM of CD3ϵ overlaps with a proline-rich sequence (PRS) that interacts with NCK [36] [100].
Molecule type definition:
`TCR(epitope,Y149_D~0~P,Y171_G~0~P,Y111~0~P,Y123~0~P,fynbind,PRS_E,Y188_E~0~P,Y199_E~0~P)`
`Y111` and `Y123` are sites in the ζ chain. Sites and motifs in the δ, ϵ, and γ chains are designated with `D`, `E`, and `G`, respectively. The region of the TCR that interacts with FYN is designated `fynbind`.

3. **CD28**
In addition to ligation of the TCR, ligation of a co-receptor such as CD28 is needed for T-cell activation. CD28 is a homodimeric glycoprotein composed of two identical disulfide-linked chains [101] that can interact with ligands of the B7 family [102, 103, 104].
Molecule type definition:
`CD28(epitope,PRS1,PRS2)`
The component `epitope` represents the extracellular region that is bound by the α-CD28 antibody. The components `PRS1` and `PRS2` represent the distinct PRS motifs that bind LCK and ITK, respectively.

4. **LCK**
LCK is a Src-family kinase (SFK). Like other members of this family, it is regulated by phosphorylation at two tyrosine residues [105]. Autoinhibition results from phosphorylation of the C-terminal tyrosine residue (Y505) by CSK [106] and formation of an intramolecular bond with the SH2 domain [107, 108]. For simplicity, we omit intramolecular binding and take LCK phosphorylated at Y505 to be autoinhibited. A second inhibitory bond involves the SH3 domain [109, 110, 111]. Phosphorylation of a tyrosine in the activation loop (Y394; this site is at position 394 in other LCK isoforms) increases kinase activity by inducing an open conformation [25] [107]. An additional site of phosphorylation is found in the LCK SH2 domain (Y192), which reduces

the affinity of this domain for its phosphotyrosine binding partners [26]. LCK is the principal kinase responsible for phosphorylation of sites in the TCR. It also phosphorylates ZAP70 at an activating site [96]. LCK is associated with the plasma membrane through palmitoyl and myristoyl modifications [112].

Molecule type definition:

`LCK(SH2,SH3,Y192~0~P,Y394~0~P,Y505~0~P)`

The PTK domain is omitted because its action is treated implicitly.

5. **FYN**

   FYN is an SFK that associates with TCR subunits via its unique N-terminal domain [113]. It can phosphorylate Y291 in WAS [114] .

   Molecule type definition:

   `FYN(unique,PTK)`

6. **ITK**

   ITK is a Tec-family tyrosine kinase that regulates Ca^{2+} mobilization and actin polymerization [115, 116, 117]. ITK associates with CD28 [118, 119, 120], and undergoes stimulated tyrosine phosphorylation [118] and an increase in kinase activity [120].

   Molecule type definition:

   `ITK(SH3,SH2,PTK,Y512~0~P)`

7. **PAG1**

   PAG1 is a transmembrane adaptor protein that, when phosphorylated, binds SFKs and their negative regulator, CSK. Stimulation of TCR signaling appears to induce the transient dephosphorylation of PAG1, thereby releasing CSK from its plasma membrane anchor [121]. Release of CSK from membrane colocalization with LCK may facilitate and/or sustain the activation of LCK and FYN [122].

   Molecule type definition:

   `PAG1(Y163~0~P,Y317~0~P)`

   SFKs interact with multiple tyrosine residues in PAG1, of which we take Y163 to be representative.

8. **CSK**

   CSK phosphorylates the inhibitory tyrosine of SFKs (e.g., Y505 in LCK), maintaining them in an inactive state [106, 108, 123, 124]. Binding of CSK to PAG1 upregulates the kinase activity of CSK [125].

   Molecule type definition:

   `CSK(SH2)`

9. **ZAP70**

   ZAP70 (zeta-associated protein of 70 kDa) is a Syk-family PTK that becomes phosphorylated and activated following TCR activation. It contains two tandem SH2 domains that bind dually-phosphorylated ITAMs in the TCR [126, 127, 128, 129]. It is activated through phosphorylation of Y493 [130].

   Molecule type definition:

   `ZAP70(SH2,PTK,Y493~0~P)`

10. **PTPN6**

    PTPN6, or SHP-1, is a PTP with two SH2 domains [131]. Its most well-characterized substrate is the activation loop phosphotyrosine of SFKs, such as Y394 in LCK [39]. Y566 in PTPN6 is another known substrate of LCK [132]. pY566 interacts with the SH2 domain of LCK [32] and phosphorylation of Y566 may enhance the enzymatic activity of PTPN6 [40].

Molecule type defintion:

`PTPN6(SH2,Y566~0~P,PTP)`

The two SH2 domains are lumped as a single SH2 for simplicity.

11. **DOK1**

DOK1 has a primarily inhibitory role in TCR signaling [133]. Phosphorylated Y449 in DOK1 is a CSK binding site [30] [134]. This site was detected in experiments and is included in the model, but for simplicity, its interaction with CSK is not included because CSK can also be recruited to PAG1.

Molecule type defintion:

`DOK1(Y449~0~P)`

12. **DOK2**

Like DOK1, DOK2 is a negative regulator of TCR signaling [133]. DOK2 binds RASA1 (p120 RasGAP) [135], which is a negative regulator of RAS signaling. pY299 is a RasGAP binding site [136].

Molecule type defintion:

`DOK2(Y299~0~P)`

13. **LAT**

LAT (linker for activation of T cells) is a transmembrane adaptor protein with no enzymatic activity [137, 138, 139, 140]. Upon TCR activation, LAT is phosphorylated on five conserved tyrosine residues, at least three of which are essential for TCR signaling [141]. These phosphotyrosines serve as docking sites for SH2 domain-containing proteins including phospholipase Cγ1 (PLCγ1) [142] and GRAP2 (GADS) [143]. These proteins do not undergo normal phosphorylation in LAT-deficient cells [144].

Molecule type definition:

`LAT(Y132~0~P,Y191~0~P)`

14. **PLCG1**

PLCG1, or PLCγ1, cleaves phosphatidylinositol 4,5-bisphosphate, generating the second messengers diacyl glycerol (DAG) and inositol 1,4,5-trisphosphate (IP_3) [145, 146]. IP_3 binds to receptors on the endoplasmic reticulum, leading to release of Ca^{2+} [147]. ITK phosphorylates PLCG1 on Y783, which is important for activation of PLCG1 enzymatic activity [117, 148, 149]. PLCG1 binds to phosphorylated LAT [143] through its N-terminal SH2 domain [150]. PLCG1 has two SH2 domains; only the N-terminal domain is considered in the model.

Molecule type definition:

`PLCG1(SH2,SH3,Y783~0~P)`

15. **LCP2**

LCP2, or SLP-76 (SH2 domain-containing leukocyte phosphoprotein of 76 kDa), is an adaptor protein essential for TCR signaling [151, 152]. ZAP70 phosphorylates LCP2 on multiple tyrosine residues, of which Y113 and Y128 can interact with NCK [153, 154]. LCP2 also contains an RxxK motif that binds the C-terminal SH3 domain of GRAP2 [155, 156].

Molecule type definition:

`LCP2(RxxK,Y113_Y128~0~P,PRS,Y145~0~P)`

Y113 and Y128 are treated as a single site for simplicity.

16. **GRAP2**

GRAP2, also known as GADS, is an adaptor protein that binds LAT and LCP2 [23]. Interactions with LAT are mediated by the GRAP2 SH2 domain [157]. The SH3 domain of GRAP2 binds an RxxK motif in LCP2 [158] with high affinity [159].

Molecule type definition:

```
GRAP2(SH2,SH3)
```

17. **NCK1/2**

NCK adaptor proteins link the TCR to the cytoskeleton. NCK1 and NCK2 are closely related and share overlapping functions [160], and thus we treat them as a single protein. NCK contains three SH3 domains and one SH2 domain, allowing it to interact with a large number of binding partners [160, 161]. Its N-terminal (first) SH3 domain can bind the PRS in CD3ϵ, which overlaps the CD3ϵ ITAM. This interaction can occur prior to ITAM phosphorylation [35]. The C-terminal (third) SH3 domain of NCK binds WAS [162] and the SH2 domain of NCK binds phosphorylated LCP2 [154].

Molecule type defintiion:

```
NCK(SH3_1,SH3_3,SH2)
```

18. **WAS**

WAS, Wiskott-Aldrich syndrome protein (WASP), is essential for regulation of the actin cytoskeleton, and mutations in this protein can lead to Wiskott-Aldrich syndrome, which is associated with abnormal lymphocyte morphology and defective TCR signaling [163, 164, 165, 166, 167, 168]. The C-terminal region of WAS interacts with the ARP2/3 complex [169]. Phosphorylation of WAS Y291 contributes to activation of WAS, potentially by destabilizing an autoinhibited conformation [170]. WAS Y291 is a substrate of several kinases, including FYN [114]. WAS is recruited to TCR signaling complexes by binding to NCK. The central proline-rich region of WAS is bound primarily by the C-terminal SH3 domain of NCK, although the other SH3 domains of NCK can also contribute cooperative interactions [171, 172]; for simplicity, these cooperative interactions are not included in the model.

Molecule type defintiion:

```
WAS(PRS,Y291~0~P)
```

Interactions in Model

Receptor stimulation

1. **CD28 cross-linking.** Ligand-induced clustering of CD28 complexes was modeled as follows:

a.
```
Lig1(aCD28,aCD28) + CD28(epitope) <-> Lig1(aCD28!1,aCD28).CD28(epitope!1) kfl,krl
```
b.
```
Lig1(aCD28!1,aCD28).CD28(epitope!1) + CD28(epitope) <->
  Lig1(aCD28!1,aCD28!2).CD28(epitope!1).CD28(epitope!2) kfl_m,krl
```

The first rule represents initial binding and the second rule represents cross-linking.

2. **CD3 cross-linking.** Ligand-induced clustering of TCR/CD3 complexes was modeled as follows:

a.
```
Lig3(aCD3,aCD3) + TCR(epitope) <-> Lig3(aCD3!1,aCD3).TCR(epitope!1) kfl,krl
```
b.
```
Lig3(aCD3!1,aCD3).TCR(epitope!1) + TCR(epitope) <->
  Lig3(aCD3!1,aCD3!2).TCR(epitope!1).TCR(epitope!2) kfl_m,krl
```

The first rule represents initial binding and the second represents cross-linking.

3. **CD3/CD28 cross-linking.** Ligand-induced co-clustering of TCR and CD28 was modeled as follows:

   a. `Lig2(aCD28,aCD3) + CD28(epitope) <-> Lig2(aCD28!1,aCD3).CD28(epitope!1) kfl,krl`
   b. `Lig2(aCD28!1,aCD3).CD28(epitope!1) + TCR(epitope) <->`
      `Lig2(aCD28!1,aCD3!2).CD28(epitope!1).TCR(epitope!2) kfl_m,krl`
   c. `Lig2(aCD28,aCD3) + TCR(epitope) <-> Lig2(aCD28,aCD3!1).TCR(epitope!1) kfl,krl`
   d. `Lig2(aCD28,aCD3!1).TCR(epitope!1) + CD28(epitope) <->`
      `Lig2(aCD28!2,aCD3!1).TCR(epitope!1).CD28(epitope!2) kfl_m,krl`

   Rules a and c represent initial binding. Rules b and d represent crosslinking.

Recruitment of signaling proteins to receptors

4. **LCK binds CD28.** The SH3 domain of LCK binds the C-terminal PRS (^{208}PYAP) in CD28. CD28 signaling requires the presence of proline residues in CD28 as well as the SH3 domain of LCK [173]. Cross-linking of CD28 is followed by an increase in LCK kinase activity [118, 119, 174]. The interaction of LCK and CD28 is modeled using the following rule:

   ```
   LCK(SH3) + CD28(PRS1) <-> LCK(SH3!1).CD28(PRS1!1) kfLckCd28,krLckCd28
   ```

5. **ITK binds CD28.** The SH3 domain of ITK binds the N-terminal PRS (^{196}PRRP) in CD28 [120, 175, 176]. CD28 ligation results in phosphorylation of ITK [118].

   ```
   CD28(PRS2) + ITK(SH3) <-> CD28(PRS2!1).ITK(SH3!1) kfITKCd28, krITKCd28
   ```

6. **FYN binds TCR.** FYN constitutively associates with the TCR through its unique N-terminal region [113]. The site in the TCR that binds FYN is designated `fynbind`.

   ```
   TCR(fynbind) + FYN(unique) <-> TCR(fynbind!1).FYN(unique!1) kfTcrFyn,krTcrFyn
   ```

7. **NCK binds TCR.** The N-terminal SH3 domain of NCK binds a PRS in CD3E [161, 177]. This PRS is exposed in a fraction of CD3E molecules under basal conditions, and this fraction increases upon TCR stimulation (including stimulation with α-CD3 antibodies) [178, 179]. A ligand-induced conformational change in the TCR is implicated in exposure of the PRS [34], but for simplicity, this conformational change is not included in the model. NCK-CD3E association does not require CD3E phosphorylation [34] This interaction may be more important for responses to weak agonists than strong agonists [47].

   a. `NCK(SH3_1) + TCR(Y188_E~0,PRS_E) <-> NCK(SH3_1!1).TCR(Y188_E~0,PRS_E!1) kfWasNck,krWasNck`
   b. `NCK(SH3_1!1).TCR(Y188_E~P,PRS_E!1) -> NCK(SH3_1) + TCR(Y188_E~P,PRS_E) 1e5*krWasNck`

   The first rule represents interactions between NCK and a TCR that is unphosphorylated at Y188 of CD3E. The second rule represents dissociation of NCK induced by phosphorylation of Y188

[36]. We assume that phosphorylation of Y188 takes place even when NCK is bound (i.e., phosphorylation is not inhibited) because the phosphorylation dynamics of this site is comparable to that of other ITAMs in our data (see Supplementary Fig. S6).

8. **ZAP70 binds phosphorylated Y111 in the ζ chain of TCR.** ZAP70 has two SH2 domains that bind phosphorylated ITAM residues in the TCR [130]. In the model, the two SH2 domains are treated as a single site. Binding of ZAP70 to phosphorylated pY111 is modeled as follows:

```
TCR(Y111~P) + ZAP70(SH2) <-> TCR(Y111~P!1).ZAP70(SH2!1) kfZapTcr,krZapTcr
```

9. **ZAP70 binds phosphorylated Y123 in the ζ chain of TCR.** ZAP70 has two SH2 domains that bind phosphorylated ITAM residues in the TCR [130]. In the model, the two SH2 domains are treated as a single site. Binding of ZAP70 to phosphorylated pY123 is modeled as follows:

```
TCR(Y123~P) + ZAP70(SH2) <-> TCR(Y123~P!1).ZAP70(SH2!1) kfZapTcr,krZapTcr
```

10. **ZAP70 binds phosphorylated Y199 in the ϵ chain of TCR.** In addition to binding ITAMs in the TCR ζ chains, ZAP70 is also capable of binding the CD3E ITAM. [127]. Binding of ZAP70 to phosphorylated pY199 is modeled as follows:

```
TCR(Y199_E~P) + ZAP70(SH2) <-> TCR(Y199_E~P!1).ZAP70(SH2!1) kfZapCd3e,krZapCd3e
```

11. **ZAP70 binds phosphorylated Y188 in the ϵ chain of TCR.** In addition to binding ITAMs in the TCR ζ chains, ZAP70 is also capable of binding the CD3E ITAM. [127]. Binding of ZAP70 to phosphorylated pY188 is modeled as follows:

```
TCR(Y188_E~P) + ZAP70(SH2) <-> TCR(Y188_E~P!1).ZAP70(SH2!1) kfZapCd3e,krZapCd3e
```

12. **PTPN6 binds phosphorylated Y149 in CD3D.** PTPN6 (SHP-1) contains two SH2 domains that have been found to bind phosphotyrosines in some cell-surface immunoreceptors [180]. SH2 domain binding disrupts an autoinhibitory intramolecular bond and contributes to PTPN6 activation [181]. Our data indicates that PTPN6 is phosphorylated rapidly, with similar dynamics to sites in proteins known to bind the TCR, such as Y493 in ZAP70 (see Figs. 2, 3, and Supplementary Fig. S6). To capture fast recruitment of PTPN6 to the membrane, we assume that it binds phosphotyrosines in the TCR δ and γ ITAMs. We note that interaction of PTPN6 with these sites has not been demonstrated. However, given the large number of phosphosites in the TCR, we take these sites to represent phosphotyrosines that may be bound by PTPN6 without creating competition with ZAP70. PTPN6 may be recruited through other interactions. For example, PTPN6 has recently been found to interact with CRKL [182]. It can also bind the SH2 domain of LCK (see Interaction 24 below); however, this interaction requires phosphorylation of PTPN6 Y566 and therefore cannot account for recruitment of unphosphorylated PTPN6. Recruitment of unphosphorylated PTPN6 is needed for the level of phosphorylated PTPN6 to increase quickly upon stimulation of TCR/CD28 signaling. A role for PTPN6 is supported by a direct test of a model prediction via RNAi knockdown (see Fig. 3) as well as our finding that Y192 in LCK is a direct substrate of PTPN6, at least *in vitro* (Fig. S9).

```
PTPN6(SH2) + TCR(Y149_D~P) <-> PTPN6(SH2!1).TCR(Y149_D~P!1) kfPtpTcr,krPtpTcr
```

13. **PTPN6 binds phosphorylated Y171 in CD3G** See note on interaction 12.

```
PTPN6(SH2) + TCR(Y171_G~P) <-> PTPN6(SH2!1).TCR(Y171_G~P!1) kfPtpTcr,krPtpTcr
```

Receptor phosphorylation

LCK phosphorylates ITAMs in TCR subunits [96, 183, 184, 185, 186, 187, 188, 189, 190, 191, 192].

14. **LCK phosphorylates Y188 in CD3E (CD3ϵ).**

```
LCK(SH3!1,Y505~0).CD28(PRS1!1,epitope!2).Lig2(aCD28!2,aCD3!3).TCR(epitope!3,Y188_E~0) ->
LCK(SH3!1,Y505~0).CD28(PRS1!1,epitope!2).Lig2(aCD28!2,aCD3!3).TCR(epitope!3,Y188_E~P) kpLckCd3e1
```

15. **LCK phosphorylates Y199 in CD3E (CD3ϵ).**

```
LCK(SH3!1,Y505~0).CD28(PRS1!1,epitope!2).Lig2(aCD28!2,aCD3!3).TCR(epitope!3,Y199_E~0) ->
LCK(SH3!1,Y505~0).CD28(PRS1!1,epitope!2).Lig2(aCD28!2,aCD3!3).TCR(epitope!3,Y199_E~P) kpLckCd3e2
```

16. **LCK phosphorylates Y149 in CD3D (CD3δ).**

```
LCK(SH3!1,Y505~0).CD28(PRS1!1,epitope!2).Lig2(aCD28!2,aCD3!3).TCR(epitope!3,Y149_D~0) ->
LCK(SH3!1,Y505~0).CD28(PRS1!1,epitope!2).Lig2(aCD28!2,aCD3!3).TCR(epitope!3,Y149_D~P) kpLckCd3d
```

17. **LCK phosphorylates Y171 in CD3G (CD3γ).** .

```
LCK(SH3!1,Y505~0).CD28(PRS1!1,epitope!2).Lig2(aCD28!2,aCD3!3).TCR(epitope!3,Y171_G~0) ->
LCK(SH3!1,Y505~0).CD28(PRS1!1,epitope!2).Lig2(aCD28!2,aCD3!3).TCR(epitope!3,Y171_G~P) kpLckCd3g
```

18. **LCK phosphorylates Y111 in CD247 (TCRζ).**

```
LCK(SH3!1,Y505~0).CD28(PRS1!1,epitope!2).Lig2(aCD28!2,aCD3!3).TCR(epitope!3,Y111~0) ->
LCK(SH3!1,Y505~0).CD28(PRS1!1,epitope!2).Lig2(aCD28!2,aCD3!3).TCR(epitope!3,Y111~P) kpLckTcrz1
```

19. **LCK phosphorylates Y123 in CD247 (TCRζ).**

```
LCK(SH3!1,Y505~0).CD28(PRS1!1,epitope!2).Lig2(aCD28!2,aCD3!3).TCR(epitope!3,Y123~0) ->
LCK(SH3!1,Y505~0).CD28(PRS1!1,epitope!2).Lig2(aCD28!2,aCD3!3).TCR(epitope!3,Y123~P) kpLckTcrz1
```

Interactions among receptor-proximal signaling proteins

20. **LCK *trans*-phosphorylates LCK.** LCK can be activated by autophosphorylation of Y394 in the kinase domain [193, 194, 195], which occurs after CD28 binding [196]. Mutations of this site reduce kinase activity [197, 198, 199, 200].

    a.
    ```
    LCK(SH3!1,Y505~P).CD28(PRS1!1,epitope!2).Lig1(aCD28!2,aCD28!3).
    CD28(epitope!3,PRS1!4).LCK(SH3!4,Y394~0) ->
    LCK(SH3!1,Y505~P).CD28(PRS1!1,epitope!2).Lig1(aCD28!2,aCD28!3).
    CD28(epitope!3,PRS1!4).LCK(SH3!4,Y394~P) kpLckLck1
    ```
    b.
    ```
    LCK(SH3!1,Y505~0).CD28(PRS1!1,epitope!2).Lig1(aCD28!2,aCD28!3).
    CD28(epitope!3,PRS1!4).LCK(SH3!4,Y394~0) ->
    LCK(SH3!1,Y505~0).CD28(PRS1!1,epitope!2).Lig1(aCD28!2,aCD28!3).
    CD28(epitope!3,PRS1!4).LCK(SH3!4,Y394~P) kpLckLck2
    ```

    The first rule characterizes phosphorylation by autoinhibited LCK (phosphorylated on the inhibitory tyrosine 505), and the second rule characterizes phosphorylation by activated LCK (unphosphorylated on tyrosine 505).

21. **LCK phosphorylates ITK Y512.** Y512 is located in the activation loop of the ITK kinase domain [201]; phosphorylation of this residue increases ITK activity. Substitution with phenylalanine causes a large reduction in activity of the kinase [202]. LCK is needed for full activation of ITK [119, 203, 204].

    a.
    ```
    LCK(SH3!1,Y505~P).CD28(PRS1!1,epitope!2).Lig1(aCD28!2,aCD28!3).
    CD28(epitope!3,PRS2!4).ITK(SH3!4,Y512~0) ->
    LCK(SH3!1,Y505~P).CD28(PRS1!1,epitope!2).Lig1(aCD28!2,aCD28!3).
    CD28(epitope!3,PRS2!4).ITK(SH3!4,Y512~P) kpLckItk1
    ```
    b.
    ```
    LCK(SH3!1,Y505~0).CD28(PRS1!1,epitope!2).Lig1(aCD28!2,aCD28!3).
    CD28(epitope!3,PRS2!4).ITK(SH3!4,Y512~0) ->
    LCK(SH3!1,Y505~0).CD28(PRS1!1,epitope!2).Lig1(aCD28!2,aCD28!3).
    CD28(epitope!3,PRS2!4).ITK(SH3!4,Y512~P) kpLckItk2
    ```

    The first rule characterizes phosphorylation by autoinhibited LCK (phosphorylated on the inhibitory tyrosine 505), and the second rule characterizes phosphorylation by activated LCK (unphosphorylated on tyrosine 505).

22. **LCK phosphorylates ZAP70 Y493.** Y493 is located in the activation loop of ZAP70, which can be phosphorylated by SFKs [205]. Phosphorylation of Y493 is necessary for ZAP70 catalytic activity [206, 207]. Mutation of this residue results in markedly reduced tyrosine phosphorylation of ZAP70 and other signaling proteins [207, 208].

    a.
    ```
    TCR(epitope!3,Y111~P!1).ZAP70(Y493~0,SH2!1).Lig2(aCD28!2,aCD3!3).
    CD28(epitope!2,PRS1!4).LCK(SH3!4,Y505~0) ->
    TCR(epitope!3,Y111~P!1).ZAP70(Y493~P,SH2!1).Lig2(aCD28!2,aCD3!3).
    CD28(epitope!2,PRS1!4).LCK(SH3!4,Y505~0) kpLckZap
    ```
    b.
    ```
    TCR(epitope!3,Y123~P!1).ZAP70(Y493~0,SH2!1).Lig2(aCD28!2,aCD3!3).
    CD28(epitope!2,PRS1!4).LCK(SH3!4,Y505~0) ->
    ```

```
TCR(epitope!3,Y123~P!1).ZAP70(Y493~P,SH2!1).Lig2(aCD28!2,aCD3!3).
CD28(epitope!2,PRS1!4).LCK(SH3!4,Y505~0) kpLckZap
```

c.
```
TCR(epitope!3,Y188_E~P!1).ZAP70(Y493~0,SH2!1).Lig2(aCD28!2,aCD3!3).
CD28(epitope!2,PRS1!4).LCK(SH3!4,Y505~0) ->
TCR(epitope!3,Y188_E~P!1).ZAP70(Y493~P,SH2!1).Lig2(aCD28!2,aCD3!3).
CD28(epitope!2,PRS1!4).LCK(SH3!4,Y505~0) kpLckZap
```

d.
```
TCR(epitope!3,Y199_E~P!1).ZAP70(Y493~0,SH2!1).Lig2(aCD28!2,aCD3!3).
CD28(epitope!2,PRS1!4).LCK(SH3!4,Y505~0) ->
TCR(epitope!3,Y199_E~P!1).ZAP70(Y493~P,SH2!1).Lig2(aCD28!2,aCD3!3).
CD28(epitope!2,PRS1!4).LCK(SH3!4,Y505~0) kpLckZap
```

Rules a, b, c, and d represent phosphorylation of ZAP70 that is bound to CD247 pY111, C247 pY123, CD3E pY188, and CD3E pY199, respectively.

23. **LCK phosphorylates PTPN6 Y566.** PTPN6 is phosphorylated following TCR stimulation, and Y566 is a substrate of LCK [32].

a.
```
LCK(SH3!1,Y505~P).CD28(PRS1!1,epitope!2).Lig2(aCD28!2,aCD3!3).
TCR(epitope!3,Y149_D~P!4).PTPN6(SH2!4,Y566~0) ->
LCK(SH3!1,Y505~P).CD28(PRS1!1,epitope!2).Lig2(aCD28!2,aCD3!3).
TCR(epitope!3,Y149_D~P!4).PTPN6(SH2!4,Y566~P) kpLckPtp1
```

b.
```
LCK(SH3!1,Y505~0).CD28(PRS1!1,epitope!2).Lig2(aCD28!2,aCD3!3).
TCR(epitope!3,Y149_D~P!4).PTPN6(SH2!4,Y566~0) ->
LCK(SH3!1,Y505~0).CD28(PRS1!1,epitope!2).Lig2(aCD28!2,aCD3!3).
TCR(epitope!3,Y149_D~P!4).PTPN6(SH2!4,Y566~P) kpLckPtp2
```

c.
```
LCK(SH3!1,Y505~P).CD28(PRS1!1,epitope!2).Lig2(aCD28!2,aCD3!3).
TCR(epitope!3,Y171_G~P!4).PTPN6(SH2!4,Y566~0) ->
LCK(SH3!1,Y505~P).CD28(PRS1!1,epitope!2).Lig2(aCD28!2,aCD3!3).
TCR(epitope!3,Y171_G~P!4).PTPN6(SH2!4,Y566~P) kpLckPtp1
```

d.
```
LCK(SH3!1,Y505~0).CD28(PRS1!1,epitope!2).Lig2(aCD28!2,aCD3!3).
TCR(epitope!3,Y171_G~P!4).PTPN6(SH2!4,Y566~0) ->
LCK(SH3!1,Y505~0).CD28(PRS1!1,epitope!2).Lig2(aCD28!2,aCD3!3).
TCR(epitope!3,Y171_G~P!4).PTPN6(SH2!4,Y566~P) kpLckPtp2
```

Rules a and c characterize phosphorylation by autoinhibited LCK (phosphorylated on the inhibitory tyrosine 505), and Rules b and d characterize phosphorylation by activated LCK (unphosphorylated on tyrosine 505). Rules a and b differ from Rules c and d in that PTPN6 is taken to be associated with CD3D in the former rules and with CD3G in the latter rules.

24. **LCK binds PTPN6.** Y566 is located in a consensus sequence for LCK binding. Mutation of this site to phenylalanine abolishes interactions between LCK and PTPN6 [32].

a.
```
LCK(SH2,Y192~0) + PTPN6(Y566~P) <-> LCK(SH2!1,Y192~0).PTPN6(Y566~P!1) kfLckPtp,krLckPtp
```

b.
```
LCK(SH2,Y192~P) + PTPN6(Y566~P) <-> LCK(SH2!1,Y192~P).PTPN6(Y566~P!1) kfLckPtp2,krLckPtp2
```

The first rule represents binding to LCK that is unphosphorylated at Y192. The second rule represents binding to LCK that is phosphorylated at Y192, which reduces the affinity of the

LCK SH2 domain [26].

Negative regulation by PAG1/CSK

25. **CSK binds PAG1.** pY317 in PAG binds the SH2 domain of CSK [209].

```
PAG1(Y317~P) + CSK(SH2) <-> PAG1(Y317~P!1).CSK(SH2!1) kfPagCsk,krPagCsk
```

26. **LCK binds PAG1.** pY163 and pY181 in PAG1 are binding sites for SFKs, and these two sites may have redundant functions [31]. Both of these sites are dephosphorylated in our data. For simplicity, we consider only one of them in our model.

```
PAG1(Y163~P) + LCK(SH2) <-> PAG1(Y163~P!1).LCK(SH2!1) kfPagLck,krPagLck
```

27. **LCK phosphorylates PAG1 Y317.** PAG1 is constitutively phosphorylated in the absence of stimulation. Tyrosine residues in PAG1 are substrates of SFKs [210].

```
PAG1(Y317~0,Y163~P!1).LCK(SH2!1) -> PAG1(Y317~P,Y163~P!1).LCK(SH2!1) kpLckPag
```

28. **CSK phosphorylates LCK.** CSK phosphorylates LCK at Y505, which facilitates formation of an inhibitory intramolecular bond. We assume that CSK-mediated phosphorylation of LCK occurs in *cis*, i.e., when both molecules are bound to the same PAG1 molecule [18].

```
PAG1(Y317~P!2,Y163~P!1).LCK(SH2!1,Y505~0).CSK(SH2!2) ->
PAG1(Y317~P!2,Y163~P!1).LCK(SH2!1,Y505~P).CSK(SH2!2) kpCskLck
```

Dephosphorylation by PTPN6

29. **PTPN6 dephosphorylates LCK Y192.** Y192 is located within the LCK SH2 domain, and phosphorylation of this site reduces affinity of the SH2 domain for phosphotyrosines [26]. LCK Y192 is rapidly dephosphorylated following TCR/CD28 co-stimulation (see Figs. 2, 3, and Supplementary Fig. S6). Knockdown of PTPN6 results in increased phosphorylation of LCK Y192 (see Fig. 3). Thus, we hypothesize that Y192 is a substrate of PTPN6. We note that a proline residue is located at +3 position C-terminal to Y192 (Y(ph)ISPR), which is also found in several reported PTPN6 and PTPN11 substrates [45]. In an *in vitro* phosphatase assay, we found that PTPN6 is able to directly dephosphorylate Y192 in LCK (Fig. S9).

a.
```
LCK(SH3!1,Y192~P).CD28(PRS1!1,epitope!2).Lig2(aCD28!2,aCD3!3).
TCR(epitope!3,Y149_D~P!4).PTPN6(SH2!4,Y566~P!?) ->
LCK(SH3!1,Y192~0).CD28(PRS1!1,epitope!2).Lig2(aCD28!2,aCD3!3).
TCR(epitope!3,Y149_D~P!4).PTPN6(SH2!4,Y566~P!?) kdpLck192
```

b. `LCK(SH3!1,Y192~P).CD28(PRS1!1,epitope!2).Lig2(aCD28!2,aCD3!3).`
`TCR(epitope!3,Y171_G~P!4).PTPN6(SH2!4,Y566~P!?) ->`
`LCK(SH3!1,Y192~0).CD28(PRS1!1,epitope!2).Lig2(aCD28!2,aCD3!3).`
`TCR(epitope!3,Y171_G~P!4).PTPN6(SH2!4,Y566~P!?) kdpLck192`

Rules a and b differ with respect to the TCR site at which PTPN6 is bound.

30. **PTPN6 dephosphorylates LCK Y394.** Dephosphorylation of LCK Y394 by PTPN6 appears to be a major negative regulatory function of PTPN6 [39].

a. `LCK(SH3!1,Y394~P).CD28(PRS1!1,epitope!2).Lig2(aCD28!2,aCD3!3).`
`TCR(epitope!3,Y149_D~P!4).PTPN6(SH2!4,Y566~P!?) ->`
`LCK(SH3!1,Y394~0).CD28(PRS1!1,epitope!2).Lig2(aCD28!2,aCD3!3).`
`TCR(epitope!3,Y149_D~P!4).PTPN6(SH2!4,Y566~P!?) kdpLck424`

b. `LCK(SH3!1,Y394~P).CD28(PRS1!1,epitope!2).Lig2(aCD28!2,aCD3!3).`
`TCR(epitope!3,Y171_G~P!4).PTPN6(SH2!4,Y566~P!?) ->`
`LCK(SH3!1,Y394~0).CD28(PRS1!1,epitope!2).Lig2(aCD28!2,aCD3!3).`
`TCR(epitope!3,Y171_G~P!4).PTPN6(SH2!4,Y566~P!?) kdpLck424`

c. `PTPN6(Y566~P!1).LCK(SH2!1,Y394~P) -> PTPN6(Y566~P!1).LCK(SH2!1,Y394~0) kdpLck394`

In Rules a and b, LCK and PTPN6 are colocalized through receptor binding. In Rule c, PTPN6 is bound directly to LCK.

31. **PTPN6 dephosphorylates PAG1 Y163.** PAG1 is observed to be rapidly dephosphorylated following TCR/CD28 co-stimulation. We hypothesize that PAG1 is a substrate of PTPN6. We found that PTPN6 knockdown (KD) resulted in a decrease in phosphorylation of ZAP70 Y493, which is a target of LCK, supporting a a positive role for PTPN6 in early TCR signaling. In the model, the positive effect of PTPN6 KD arises partly because dephosphorylation of PAG1 limits colocalization of LCK and CSK on PAG1 and CSK-mediated phosphorylation of LCK 505 (and LCK autoinhibition). LCK autoinhibition could also be limited if PTPN6 acted directly on LCK pY505. However, it seems that PTPN6-mediated dephosphorylation of pY505 is limited because of intramolecular interaction of pY505 with the LCK SH2 domain [39]. We note that PTPN11 (SHP-2), which is closely related to PTPN6, has been found to act on sites in PAG1 [211].

a. `PAG1(Y163~P) + PTPN6(SH2!2,PTP,Y566~P!?).TCR(Y149_D~P!2) ->`
`PAG1(Y163~P!1).PTPN6(SH2!2,PTP!1,Y566~P!?).TCR(Y149_D~P!2) kfPagPtp`

b. `PAG1(Y163~P) + PTPN6(SH2!2,PTP,Y566~P!?).TCR(Y171_G~P!2) ->`
`PAG1(Y163~P!1).PTPN6(SH2!2,PTP!1,Y566~P!?).TCR(Y171_G~P!2) kfPagPtp`

c. `PTPN6(PTP,SH2) + PAG1(Y163~P) -> PTPN6(PTP!1,SH2).PAG1(Y163~P!1) kfPagPtp_cyt`

d. `PAG1(Y163~P!1).PTPN6(PTP!1) -> PAG1(Y163~P) + PTPN6(PTP) krPagPtp`

e. `PAG1(Y163~P!1).PTPN6(PTP!1,Y566~P!?) -> PAG1(Y163~0) + PTPN6(PTP,Y566~P!?) kdpPag`

Rules a and b characterize binding of membrane-recruited PTPN6 to PAG1, Rule c characterizes binding of PTPN6 from the cytosol, Rule d characterizes dissociation, and Rule e characterizes dephosphorylation and dissociation.

32. **PTPN6 dephosphorylates DOK1 pY449.** DOK1 pY449 follows a similar time course as other sites of dephosphorylation (see Fig. 2). Thus, we hypothesize that it is a substrate of

PTPN6, although other phosphatases may be involved (see notes on Interaction 12). DOK1 has been found to be dephosphorylated by PTPN6 [212], but Y449 has not been specifically identified as a substrate.

a. `DOK1(Y449~P) + PTPN6(SH2!2,PTP).TCR(Y149_D~P!2) ->`
   `DOK1(Y449~P!1).PTPN6(SH2!2,PTP!1).TCR(Y149_D~P!2) kfDok1Ptp`
b. `DOK1(Y449~P) + PTPN6(SH2!2,PTP).TCR(Y171_G~P!2) ->`
   `DOK1(Y449~P!1).PTPN6(SH2!2,PTP!1).TCR(Y171_G~P!2) kfDok1Ptp`
c. `DOK1(Y449~P!1).PTPN6(PTP!1) -> DOK1(Y449~P) + PTPN6(PTP) krDok1Ptp`
d. `DOK1(Y449~P!1).PTPN6(PTP!1,Y566~P!?) -> DOK1(Y449~0) + PTPN6(PTP,Y566~P!?) kdpDok1`

Rules a and b characterize binding of PTPN6 to DOK1, Rule c characterizes dissociation, and Rule d characterized dephosphorylaton and dissociation.

33. **PTPN6 dephosphorylates DOK2 pY299.** DOK2 pY299 follows a similar time course as other sites of dephosphorylation (see Fig. 2 and Supplementary Fig. S6). Thus, we hypothesize that it is a substrate of PTPN6, although other phosphatases may be involved (see notes on Interaction 12).

a. `DOK2(Y299~P) + PTPN6(SH2!2,PTP).TCR(Y149_D~P!2) ->`
   `DOK2(Y299~P!1).PTPN6(SH2!2,PTP!1).TCR(Y149_D~P!2) kfDok2Ptp`
b. `DOK2(Y299~P) + PTPN6(SH2!2,PTP).TCR(Y171_G~P!2) ->`
   `DOK2(Y299~P!1).PTPN6(SH2!2,PTP!1).TCR(Y171_G~P!2) kfDok2Ptp`
c. `DOK2(Y299~P!1).PTPN6(PTP!1) -> DOK2(Y299~P) + PTPN6(PTP) krDok2Ptp`
d. `DOK2(Y299~P!1).PTPN6(PTP!1,Y566~P!?) -> DOK2(Y299~0) + PTPN6(PTP,Y566~P!?) kdpDok2`

Rules a and b characterize binding of PTPN6 to DOK2, Rule c characterizes dissociation, and Rule d characterized dephosphorylaton and dissociation.

Shortcut recruitment and phosphorylation of WAS

34. **NCK1/2 binds WAS.** NCK is needed for recruitment of WAS to regions of TCR stimulation [162]. The third SH3 domain of NCK can bind to the central proline-rich region of WAS [171, 172]. This interaction is part of both the long and short pathways for WAS activation.

`WAS(PRS) + NCK(SH3_3) <-> WAS(PRS!1).NCK(SH3_3!1) kfWasNck,krWasNck`

35. **FYN phosphorylates WAS.** WAS can be phosphorylated by SFKs [213]. WAS associates with FYN [214] and FYN deficiency is associated with a greater reduction in WAS phosphorylation than either LCK or ITK deficiency, suggesting that FYN is the primary kinase responsible for phosphorylation of WAS [114].

a. `TCR(epitope!3,fynbind!1).FYN(unique!1).Lig3(aCD3!2,aCD3!3).`
   `TCR(epitope!2,PRS_E!4).NCK(SH3_1!4,SH3_3!5).WAS(Y291~0,PRS!5) ->`

```
TCR(epitope!3,fynbind!1).FYN(unique!1).Lig3(aCD3!2,aCD3!3).
TCR(epitope!2,PRS_E!4).NCK(SH3_1!4,SH3_3!5).WAS(Y291~P,PRS!5) kpWasp
```

b.
```
LAT(Y191~P!1).GRAP2(SH2!1,SH3!2).LCP2(RxxK!2,Y113_Y128~P!3).NCK(SH2!3,SH3_3!4).
WAS(PRS!4,Y291~0) + FYN(unique!+,PTK) ->
LAT(Y191~P!1).GRAP2(SH2!1,SH3!2).LCP2(RxxK!2,Y113_Y128~P!3).NCK(SH2!3,SH3_3!4).
WAS(PRS!4,Y291~0!5).FYN(unique!+,PTK!5) kfWasFyn
```

c. `WAS(Y291~0!5).FYN(PTK!5) -> WAS(Y291~P) + FYN(PTK) kpWas`

d. `WAS(Y291~0!1).FYN(PTK!1) -> WAS(Y291~0) + FYN(PTK) krWasFyn`

The first rule characterizes *trans*-phosphorylation by FYN when WAS is recruited to the TCR via NCK (the shortcut pathway). The remaining rules characterize binding, phosphorylation, and dissociation, respectively, when WAS is recruited to the plasma membrane by LCP2 (the long pathway; see Interactions 37 and 39-42).

Signaling through downstream adaptor proteins

36. **ZAP70 phosphorylates LAT Y132.** Y132 in LAT is phosphorylated by ZAP70 and is a PLCG1 binding site [215].

    a. `ZAP70(SH2!+,PTK) + LAT(Y132~0) -> ZAP70(SH2!+,PTK!1).LAT(Y132~0!1) kfZapLat132`
    b. `ZAP70(PTK!1).LAT(Y132~0!1) -> ZAP70(PTK) + LAT(Y132~0) krZapLat`
    c. `ZAP70(PTK!1,Y493~P).LAT(Y132~0!1) -> ZAP70(PTK,Y493~P) + LAT(Y132~P) kpZapLat2`

    These rules correspond to a Michaelis-Menten reaction scheme.

37. **ZAP70 phosphorylates LAT Y191.** Y191 in LAT is required for T-cell activation and is phosphorylated by ZAP70 [215].

    a. `ZAP70(SH2!+,PTK) + LAT(Y191~0) -> ZAP70(SH2!+,PTK!1).LAT(Y191~0!1) kfZapLat191`
    b. `ZAP70(PTK!1).LAT(Y191~0!1) -> ZAP70(PTK) + LAT(Y191~0) krZapLat`
    c. `ZAP70(PTK!1,Y493~P).LAT(Y191~0!1) -> ZAP70(PTK,Y493~P) + LAT(Y191~P) kpZapLat2`

    These rules correspond to a Michaelis-Menten reaction scheme.

38. **PLCG1 binds LAT.** pY132 in LAT is a PLCG1 binding site [215, 216]. Mutation of this site abolishes PLCG1 association with LAT and also has an impact on calcium flux, which is regulated by PLCG1 activity [143]. Interactions with LAT may also disrupt an inhibitory intramolecular bond between the SH2 domain and the split Pleckstrin homology domain in PLCG1 [217].

    `PLCG1(SH2) + LAT(Y132~P) <-> PLCG1(SH2!1).LAT(Y132~P!1) kfPlcgLat,krPlcgLat`

39. **LAT binds GRAP2.** GRAP2 binds phosphorylated Y191 in LAT [216], and mutation of this site significantly reduces association between GRAP2 and LAT [143].

```
LAT(Y191~P) + GRAP2(SH2) <-> LAT(Y191~P!1).GRAP2(SH2!1) kfLatGrap,krLatGrap
```

40. **GRAP2 binds LCP2.** The SH3 domain of GRAP2 binds an RxxK motif in LCP2 with high affinity [159].

```
GRAP2(SH3) + LCP2(RxxK) <-> GRAP2(SH3!1).LCP2(RxxK!1) kfGrapLcp, krGrapLcp
```

41. **LCP2 binds NCK.** LCP2 contains YESP motifs at Y113 and Y128 that undergo phosphorylation and bind NCK. Y128 may be the more important NCK binding site [153, 154, 218]. In our model, we lump the two sites together as a single site.

```
NCK(SH2) + LCP2(Y113_Y128~P) <-> NCK(SH2!1).LCP2(Y113_Y128~P!1) kfNckLcp,krNckLcp
```

42. **ZAP70 phosphorylates LCP2 Y113/Y128.** LCP2 is a substrate of ZAP0 [219].

```
a. LAT(Y191~P!1).GRAP2(SH2!1,SH3!2).LCP2(RxxK!2,Y113_Y128~0) + ZAP70(SH2!+,PTK) ->
   LAT(Y191~P!1).GRAP2(SH2!1,SH3!2).LCP2(RxxK!2,Y113_Y128~0!3).ZAP70(SH2!+,PTK!3) kfZapLcp
b. ZAP70(PTK!1).LCP2(Y113_Y128~0!1) -> ZAP70(PTK) + LCP2(Y113_Y128~0) krZapLcp
c. LCP2(Y113_Y128~0!3).ZAP70(PTK!3,Y493~P) -> LCP2(Y113_Y128~P) + ZAP70(PTK,Y493~P) kpZapLcp2
```

These rules correspond to a Michaelis-Menten reaction scheme.

43. **PLCG1 binds LCP2.** The SH3 domain of PLCG1 interacts with the proline-rich region of LCP2, and this interaction is important for PLCG1 activation [220].

```
PLCG1(SH3) + LCP2(PRS) <-> PLCG1(SH3!1).LCP2(PRS!1) kfPlcgLcp,krPlcgLcp
```

44. **ITK binds LCP2.** Phosphorylated Y145 in LCP2 is a binding site for the SH2 domain of ITK [221]. Interactions with LCP2 may maintain ITK in an active conformation [149].

```
LCP2(Y145~P) + ITK(SH2) <-> LCP2(Y145~P!1).ITK(SH2!1) kfLcpItk,krLcpItk
```

45. **ZAP70 phosphorylates LCP2 Y145.** LCP2 is a substrate of ZAP0 [219].

```
a. LAT(Y191~P!1).GRAP2(SH2!1,SH3!2).LCP2(RxxK!2,Y145~0) + ZAP70(SH2!+,PTK) ->
   LAT(Y191~P!1).GRAP2(SH2!1,SH3!2).LCP2(RxxK!2,Y145~0!3).ZAP70(SH2!+,PTK!3) kfZapLcp
b. ZAP70(PTK!1).LCP2(Y145~0!1) -> ZAP70(PTK) + LCP2(Y145~0) krZapLcp
c. LCP2(Y145~0!3).ZAP70(PTK!3,Y493~P) -> LCP2(Y145~P) + ZAP70(PTK,Y493~P) kpZapLcp2
```

These rules correspond to a Michaelis-Menten reaction scheme.

46. **ITK phosphorylates PLCG1.** Phosphorylation of Y783 is implicated in PLCG1 activation. Y783 is phosphorylated by ITK and requires formation of a LCP2-PLCG1-ITK complex [149, 222]. Association with LAT disrupts an intramolecular bond in PLCG1 [217] and may thereby also enable phosphorylation of PLCG1 Y783.

```
PLCG1(SH3!1,SH2!+,Y783~0).LCP2(PRS!1,Y145~P!2).ITK(SH2!2) ->
PLCG1(SH3!1,SH2!+,Y783~P).LCP2(PRS!1,Y145~P!2).ITK(SH2!2) kpPlcg
```

This rule specifies that PLCG1 is phosphorylated when it is part of a ternary complex with LCP2 and ITK. Additionally, we assume that its SH2 domain must be bound (to LAT pY132) for Y783 to be accessible.
